# Molecular and Cellular Mechanisms of Propolis and Its Polyphenolic Compounds against Cancer

**DOI:** 10.3390/ijms231810479

**Published:** 2022-09-09

**Authors:** Nada Oršolić, Maja Jazvinšćak Jembrek

**Affiliations:** 1 Division of Animal Physiology, Faculty of Science, Rooseveltov trg 6, 10000 Zagreb, Croatia; 2 Division of Molecular Medicine, Ruđer Bošković Institute, Bijenička 54, 10000 Zagreb, Croatia; 3 School of Medicine, Catholic University of Croatia, Ilica 242, 10000 Zagreb, Croatia

**Keywords:** cancer, propolis, polyphenolic/flavonoid compounds, molecular targets, chemoprevention, epigenetic and genetic mechanisms, cancer therapy

## Abstract

In recent years, interest in natural products such as alternative sources of pharmaceuticals for numerous chronic diseases, including tumors, has been renewed. Propolis, a natural product collected by honeybees, and polyphenolic/flavonoid propolis-related components modulate all steps of the cancer progression process. Anticancer activity of propolis and its compounds relies on various mechanisms: cell-cycle arrest and attenuation of cancer cells proliferation, reduction in the number of cancer stem cells, induction of apoptosis, modulation of oncogene signaling pathways, inhibition of matrix metalloproteinases, prevention of metastasis, anti-angiogenesis, anti-inflammatory effects accompanied by the modulation of the tumor microenvironment (by modifying macrophage activation and polarization), epigenetic regulation, antiviral and bactericidal activities, modulation of gut microbiota, and attenuation of chemotherapy-induced deleterious side effects. Ingredients from propolis also ”sensitize“ cancer cells to chemotherapeutic agents, likely by blocking the activation of the transcription factor nuclear factor kappa-light-chain-enhancer of activated B cells (NF-κB). In this review, we summarize the current knowledge related to the the effects of flavonoids and other polyphenolic compounds from propolis on tumor growth and metastasizing ability, and discuss possible molecular and cellular mechanisms involved in the modulation of inflammatory pathways and cellular processes that affect survival, proliferation, invasion, angiogenesis, and metastasis of the tumor.

## 1. Introduction

Despite the tremendous research efforts and rapid development of novel therapies and miracle drugs, cancer is the second leading cause of death in the world. As a hyperproliferative disorder, cancer induces morphological transformation, disturbs apoptotic signaling, and drives uncontrolled proliferation, invasion, angiogenesis, and metastasis spreading. It also includes a number of genetic and epigenetic modifications that affect the regulation of cell proliferation and survival, such as deregulated CpG dinucleotide methylation and aberrant histone acetylation that can impair the immunogenic potential of cancer cells. During the multistage processes of tumor formation, cancer cells acquire specific properties that differentiate them from healthy cells: resistance to growth inhibition, growth-factor independent proliferation, ongoing replication, escape from apoptosis, migration, invasion, formation of metastasis, and support of angiogenesis [1,2,3,4,5,6,7].

It is estimated that the number of new cancer cases reached 19.3 million, together with 10 million deaths, in 2020. The most diagnosed cancer is female breast cancer (11.7% of all cases) and is closely followed by lung cancer (11.4%). Prostate cancer (7.3%) and non-melanoma of skin (6.2%) and colon cancer (6%) are the rest of the top five most commonly diagnosed cancers. Lung cancer is the most common cause of death (18% of all cancer deaths), followed by colorectal (9.4%), liver (8.3%), stomach (7.7%), and female breast (6.9%) cancers [2]. According to Aggarwal et al. [4], most of all cancer cases (90–95%) are attributable to lifestyle, while 5–10% are associated with faulty genes. So far, more than 500 different genes have been identified contributing to tumor development and progression, suggesting that multitarget drugs would be advantageous as a treatment option in cancer therapy. This brings us to honeybee products such as propolis and its polyphenolic compounds, which are able to target multiple gene products and could be considered as promising candidates in cancer prevention and treatment.

The primary goal of current research efforts is largely devoted to the discovery of natural compounds and synthetic chemicals that can be useful in the prevention of cancer and/or as cancer therapy. Among other health-promoting biological activities, products rich in flavonoids exert strong immunopotentiatory effects and antitumor activities. Honey, propolis, and royal jelly, all products collected by bees, contain numerous phenolic compounds that have beneficial effects on human health [5,6,7,8]. The concentration of flavonoids, which represent the majority of phenolic bioactive molecules in honeybee products, depends on various factors, including plant species used, health of the plant, season, environmental factors, and geographical origin, among others [5,6,7,8].

Treatment with bee products (apitherapy), as an alternative medicine practice, has been used since ancient times and is increasingly appreciated as a medical support by many scientific authorities worldwide. Bee products, of which the most researched are honey, propolis, pollen, and royal jelly, are recognized as nutritious food and health products in apitherapy. Their biological effects are mostly attributed to phenolic compounds. Results of previous research suggest that honeybee products and their flavonoid components are particularly promising as antitumor [9,10,11,12,13,14,15,16,17,18,19,20], immunomodulatory [21,22,23,24,25,26,27], and radioprotective [28,29,30,31,32,33,34,35] agents. Results of epidemiological studies also support the important contribution of foodstuffs of vegetable origin in the prevention of numerous illnesses, including cancer [36,37,38,39,40].

A lot of research has investigated the chemical properties of honeybee products. Numerous isolated chemical compounds have been studied, and it appears that honeybee products represent a substantial source of antitumor compounds with antioxidant, anti-inflammatory, and immune-stimulatory properties [38,39,40,41,42,43,44,45]. The enhancement of the host immune response has been suggested as a plausible mechanism underlying the inhibition of tumor growth, without causing harm to the host. The natural antioxidants from honey, among which flavonoids are widely present, may account for this activity. Flavonoids affect proliferation, differentiation, and apoptotic death of cancer cells and may have a prominent role in cancer chemoprevention. Chemoprevention of tumors with natural compounds, including honeybee products, such as honey, propolis, pollen, and their related polyphenolic/flavonoid compounds, has been appreciated as a reliable antitumor approach capable to reduce the incidence of cancer development and its growth.

Apart from the above-mentioned biological activities (from anti-oxidative, and anti-inflammatory to antitumor), polyphenolic/flavonoid compounds are also strong inhibitors of enzymes involved in intracellular signal transduction pathways. They inhibit protein kinase C (PKC), tyrosine kinases, and lipid kinases and impact different metabolic processes, such as glycolytic enzyme activation and protein synthesis. They also block the cell cycle in the G0/G1 or G1/M phase, depending on their structure and target cells. Other established effects of flavonoids are binding to type II estrogen receptors, regulation and modulation of cell growth, and initiation of apoptosis in various cell lines and animal models [3,7,13,46,47,48,49].

Flavonoids selectively affect tumor cells and minimize the collateral damage induced by radiation and chemotherapy. They may enhance the antiproliferative effect of certain chemotherapeutics, but there is no sufficient data either on their mutual interactions or on the molecular mechanisms of action. Taking into consideration that the daily human diet includes intake of large amounts of flavonoids, it is logical that there is interest in their application and potential biological effects based on the unique chemical structure.

Propolis is one of the most researched hive products, the richest in polyphenolic/flavonoid components. It is a “golden product of the hive” with a wide range of biological activities. Based on that, in recent years, interest in the health effects of honeybee propolis has been revived. The results of many studies indicate that propolis possesses a wide spectrum of activities including antibacterial, antifungal, cytostatic, wound healing, antitumor, anti-diabetic, anti-allergic, and anti-inflammatory [36,37,38,39,40,41,42,43,44].

## 2. Propolis

Propolis (bee glue) is a resinous mixture collected by honeybees from leaf buds and tree sap. It is used by honeybees for sealing holes in honeycombs, and to smooth out the inside walls, reinforcing the structural stability of the hive and protecting the hive entrance from intruders. Raw propolis typically contains 50% plant resin, 30% beeswax, 10% essential and aromatic oil, and 5% pollen. The rest are various organic compounds, and some micro and macro minerals. Overall, in propolis from different geographical areas, over 800 compounds have been identified. These include phenolic acids, flavonoids (flavones, flavanones, flavonols, dihydroflavonols, and chalcones), terpenes, lignans, amino acids, fatty acids, vitamins, and minerals [5,6,7,8,9,50,51]. Besides geographical location, the chemical profile of propolis also depends on plant sources and bee species [5,6,7,8,9,51,52,53,54]. The main chemical components of propolis are fatty and aliphatic acids (24–26%), flavonoids (18–20%), and sugars (15–18%). Compounds that are present in less than 10% are aromatic acids (5–10%), esters (2–6%), vitamins (2–4%), alcohol and terpenes (2–3.3%), microelements (0.5–2.0%), and others (21–27%) [51,52,53,54].

Regarding elements, about 30 have been found, of which calcium, manganese, zinc, copper, silicon, iron, and aluminum are the most abundant [52,53,54]. Vitamins that have been found in propolis include B-group vitamins (B1, B2, B6, niacin, and folate), vitamins C, D, and E, and pro-vitamin A (β-carotene). Small amounts of enzymes, mostly originating from the bee glandular secretion and possibly from pollen, are also present: α-and β-amylase, α- and β-lactamase, maltase, esterase, and transhydrogenase [6,7,8,9,51,52,53,54]. The total protein content in the ethanol extract of propolis (EEP) is estimated to be 2.8%, on average [6,7,8,51,52,53,54,55]. Free amino acids (about 17) are present in low amounts [6,7,8]. In recent years, pyroglutamic acid, an amino acid derivative found in bees, has also been identified in propolis. Poly-, di-, and monosaccharides are also present: saccharose, glucose, fructose, rhamnose, ribose, talose, and gulose [6,7,8,9,51,52,53,54,55].

There are several types of propolis according to the geographical origin and collecting season (Table 1). The major component of propolis from North America, Europe, and non-tropical Asia is bud resin from poplar trees (*Populus* species). Different samples of poplar-type propolis have comparable chemical profiles. The principal components are phenolics, including flavonoid aglycones, aromatic acids, and their esters. Brazilian green (Alecrim) propolis is collected from *Baccharis dracunculifolia* (Asteraceae) and specifically contains prenylated derivatives of p-coumaric acid and o-hydroxy-acetophenone. Flavonoids, diterpenes, and lignans, different from those in European propolis, may also be found in Brazilian propolis. Red propolis is characteristic of Cuba, where its botanical origin is *Clusia nemorosa* (Clusiaceae), and of Venezuela, where it is collected from *C. scrobiculata*. Characteristic components that differentiate Cuban propolis from both European and Brazilian propolis are polyprenylated benzophenones. Propolis produced in the Pacific region contains geranyl flavanones, which are also found in propolis from the African regions [51].

Regardless of its plant source and chemical profile, propolis consistently exerts antimicrobial, antioxidative, immunomodulatory, anti-inflammatory, anti-allergic, derma-protective, laxative, anti-diabetic, anti-angiogenic, and antitumor activity [8,9,10,11,12,13]. The antimicrobial activity is attributed to flavonoids such as pinocembrin, galangin, pinobanksin, pinobanksin-3-acetate, and caffeic acid esters. Propolis with powerful antioxidant activity contains kaempferol, caffeic acid, and phenethyl caffeate. Flavonoids such as quercetin, acacetin, and naringenin, and cinnamic acid derivatives including baccharin, drupanin, and caffeic acid phenethyl ester (CAPE) are important for the anti-inflammatory activity of propolis. CAPE is the major constituent of propolis from temperate zones that exerts a broad range of biological effects, including the inhibition of nuclear factor κB (NF-κB), suppression of cell proliferation, stimulation of cell cycle arrest, and induction of apoptosis. On the other hand, components from Brazilian, Cuban, and Mexican propolis, demonstrated both pro- and anti-inflammatory effects, depending on the dose applied, which might be useful in the development of new immunomodulatory drugs [55].

The concentration of bioactive molecules in propolis may vary substantially in samples of different origins, and such differences may affect its biological activities and pharmacological effects. The characteristic compounds of propolis from different geographical origins are listed in Table 1.

The healing properties of propolis are recognized in traditional medicine since ancient times. Recent findings have revealed that the therapeutic efficacy of propolis in wound healing is related to the expression of collagen types I and III and wound matrix degradation, suggesting that propolis creates an appropriate biochemical milieu for re-epithelization [56].

Propolis and its bioactive molecules also exhibit prominent cytostatic [57,58,59], anti-carcinogenic, and antitumor effects, both in in vitro and in vivo tumor models [57,58,59,60,61]. Hence, propolis and its major components (caffeic acid, CAPE, artepillin C, quercetin, naringenin, resveratrol, galangin, genistein, and others) are considered as promising antineoplastic agents [5,46,47,48,49,58,59,60,61,62,63]. A growing amount of data from in vitro and in vivo studies has shown that flavonoids act as chemopreventive agents and increase the efficacy of chemotherapy and radiotherapy in various cancers by regulating the activity of Akt, NF-κB, cyclooxygenase (COX)-2, c-Myc, apoptotic, and other pathways. The diversity of their effects suggests that propolis and its active compounds could be a novel and multitarget therapeutic strategy in cancer therapy [3,4,5,6,7,9,10,11,12]. This approach provides new opportunities from cancer prevention to cancer treatment (Table 2).

## 3. Molecular and Cellular Targets of Propolis and Its Flavonoids in Cancer Chemoprevention

Accumulating evidence suggests that propolis and its flavonoids have a wide range of molecular targets either through direct interactions or through modulation of gene expression. Oršolić and Bašić [3] summarized knowledge of the various molecular mechanisms of chemopreventive or therapeutic action of flavonoids and other polyphenols in the prevention of chemical carcinogenesis in animal models. These molecular mechanisms can be divided into several categories: (i) mechanisms involved in the prevention of metabolic activation of carcinogens, (ii) mechanisms that inhibit proliferation of tumor cells through inactivation or downregulation of prooxidant enzymes and signal transduction enzymes, (iii) mechanisms involved in the initiation of cell death (apoptosis), and (iv) mechanisms that inhibit mitochondrial functions. When considering molecular targets relevant for cancer prevention, it has been shown that the flavonoids quercetin and kaempferol interact with the transcription factor aryl hydrocarbon receptor, which has a prominent role in the development of chemical-induced cancer [13].

Molecular targets modulated by propolis and its flavonoids include transcription factors, growth factors and their receptors, cytokines, enzymes, and genes participating in the regulation of cell proliferation, and apoptosis [3,4,5,6,7,9,10,11,12,13,14].

Growing data [3,4,5,6,7,13,14] suggest that propolis and its polyphenolic/flavonoid compounds are multitarget agents in cancer prevention. Possible molecular targets underlying effects of propolis and flavonoids on different stages of carcinogenesis, as well as protective effects against tumors, are shown in Table 2 and Table 3. As indicated, the modulation of oncogenes, tumor suppressor genes, and intracellular signaling pathways inhibits cell proliferation and induces transformation, angiogenesis, and apoptosis, which have been proposed as the key mechanisms of the chemopreventive action of diverse polyphenolic compounds.

Various flavonoids demonstrate powerful antioxidant properties in vitro and in vivo, directly scavenging a broad spectrum of reactive oxygen, nitrogen, and chlorine species, such as superoxide anion, hydroxyl radical, peroxyl radical, hypochlorous acid, and peroxynitrous acid. In addition, they act as metal ion chelators, thus reducing their prooxidant capacity via Fenton chemistry [3,41,42,43,62]. Furthermore, the antioxidant action of flavonoids is achieved through the inhibitory activity of certain enzymes that participate in ROS production (e.g., xanthine oxidase, PKC, ascorbic acid oxidase, cyclooxygenases, lipoxygenases, Na^+^/K^+^ ATPase, and cAMP phosphodiesterase), or by potentiating the action of other cellular antioxidants. By scavenging free radicals, chelating metal ions (mainly iron and copper), and stimulating endogenous antioxidant defenses, propolis and its flavonoids directly attenuate the generation of ROS, reduce lipid peroxidation and oxidative DNA damage, decrease levels of oxidized glutathione and increase the pool of reduced glutathione, restore activities of antioxidative enzymes, such as catalase, superoxide dismutase, glutathione S-transferase, and glucose 6-phosphate dehydrogenase, modulate signal transduction pathways, such as ERK-Nrf2-HO1 pathways, and regulate expression and activity of proteins involved in redox regulation such as glutamate-cysteine ligase regulatory subunit (GCLM) and thioredoxin reductase (TrxR1) [13,14,63,64,65,66,67,68,69,70].

In addition, flavonoids and other phenolic compounds may inhibit activities of telomerase [63,64,65,66,67,68], matrix metalloproteinases [3,4,5,6,7,71,72], angiotensin-converting enzyme [73], and sulfotransferase [74]. They also may interact with sirtuins [75] and cellular drug transport systems [76,77,78], compete with glucose for transport across the membrane [79,80,81], interfere with cyclin-mediated cell cycle regulation [1,2,3,4], and modulate platelet function [82]. Furthermore, isoflavones and lignans found in propolis act as phytoestrogens and modulate hormone-dependent carcinogenesis in animals [83,84,85]. Finally, it must be emphasized that flavonoids are recognized as xenobiotics, which are visible by their rapid metabolism, and their detrimental effects have been observed in vitro and in vivo [70,86,87].

### 3.1. Oxidative Stress and Tumor Development: Role of Propolis and Its Polyphenolic/Flavonoid Components

Therapeutic effects of propolis on numerous diseases, such as infections, hypertension, cardiovascular disorders, diabetes, allergies, asthma, and cancer, are assigned to its anti-oxidative, immuno-modulatory, and anti-inflammatory properties [3,8,9,10,11,12,13,14,15,16,17,34,35,36,37,38,39,40,41,42,43,88]. These diseases are accompanied by mitochondrial dysfunction and increased ROS production and the enzymatic and non-enzymatic oxidation of polyunsaturated fatty acids (PUFAs). Fenton and Haber–Weiss reactions, mainly catalyzed by iron ions, generate ^•^OH (hydroxyl radicals) from H_2_O_2_ (hydrogen peroxide) and superoxide anions (^•^O_2_^−^). During normal metabolism and NADPH oxidation, ROS/RNS (e.g., OH•, O_2_^•^^−^, nitric oxide NO, nitrogen dioxide NO_2_, and peroxyl ROO^•^ and lipid peroxyl LOO• radicals) are continuously produced, and are significantly involved in cellular aging, mutagenesis, and carcinogenesis. At physiological concentrations, ROS are important signaling molecules involved in the regulation of cell growth, the adhesion of cells toward other cells, differentiation, senescence, and apoptosis, while prolonged and enhanced ROS production is considered essential for the progression of inflammatory disease. Inflammation is a critical component of tumor progression. ROS-sensitive signaling pathways are persistently activated in many types of cancers, contributing to cell growth/proliferation, differentiation, protein synthesis, glucose metabolism, cell survival, and inflammation [89]. Oxidative DNA damage considerably contributes to mutagenesis and cancer. If unrepaired, ROS-induced DNA damage may result in mutations. Moreover, oxidative damage of oncogenes or tumor suppressor genes may accelerate cancer transformation. Thus, ROS participate in both the development and progression of cancer [90]. ROS may also regulate cell motility and tumor cell metastasis by increasing vascular permeability. Furthermore, ROS can induce intracellular signaling pathways by reversible oxidation of phosphatases, such as phosphatase and tensin homolog (PTEN) or protein tyrosine phosphatase (PTP), at cysteine residues in their active sites, or by direct oxidation of kinases such as Src. This results in the activation of several signaling pathways such as Src/PKD1-mediated activation of NF-κB, the mitogen-activated protein kinases (MAPKs) (ERK1/2, p38, and JNK) signaling pathways, and the phosphatidylinositide 3-kinases/protein kinase B (PI3K/Akt) signaling cascade. However, another mechanism by which ROS initiate cellular signaling is based on the activation of redox-regulated transcription factors such as activating protein-1 (AP-1) or forkhead transcription factors (FOXO) [89,90,91,92].

It has to be emphasized that cancer development is a multistage process that may be induced by environmental hazards, inflammatory agents, and modulators of transcription factors (e.g., NF-κB, STAT3, and AP-1) that mediate communication between cancer cells and inflammatory cells. Overexpression of proinflammatory transcription factors NF-κB, STAT3, and AP-1 results in the release of cytokines and chemokines, and pre-existing inflammation plays a critical role in immunosuppression via the STAT3 pathway, which stimulates the expression of programmed death ligands 1 and 2 (PD-L1 and PD-L2) in cancer cells and suppresses the activity of immune cells [93].

#### 3.1.1. Antioxidative Activity of Propolis and Its Polyphenolic Compounds

Numerous components of propolis, such as quercetin, caffeic acid, CAPE, p-coumaric acid, ferulic acids, and chrysin, decrease the production of pro-inflammatory molecules by inhibiting the expression of ROS-producing enzymes, such as lipoxygenases, cyclooxygenases, phospholipase A2, and nitric oxidase synthase [3,4,5,6,7]. Additionally, some propolis components repress the activation of NF-κB, and modulate MAPKs and arachidonic acids pathways by blocking their translocation to the nucleus. In almost all tumor cells, constitutive activation of NF-κB drives proliferation, and NF-κB inhibition abrogates proliferation [94].

A positive correlation has been shown between high polyphenol levels and antioxidant activity, in general, due to the chemical structure of phenolic acids and flavonoids.

As previously mentioned, flavonoids are effective radical scavengers. Their phenolic hydroxyl groups can be easily oxidized, which contributes to the antioxidant/antiradical capacity of flavonoids. The structural characteristics of flavonoids that contribute to these properties are the presence of: (i) ortho-hydroxyl on the B-ring, (ii) free hydroxyl group(s) in the B ring, (iii) C2-C3 double bond in the C-ring of the backbone, or (iv) free hydroxyl group at the C-3 or C-4′ position of the flavonoid skeleton. Another important mechanism of the antioxidative action of flavonoids relies on their interactions with redox enzymes in detoxification processes, such as NAD(P)H-quinone oxidoreductase, glutathione S-transferase, or UDP-glucuronosyl transferase, which are involved in the protection against oxidative stress [3,4,7].

#### 3.1.2. Prooxidative Activity of Propolis and Its Polyphenolic Compounds

Flavonoids may prevent the toxic effects of redox-active metals such as Fe or Cu, which can catalyze the production of ROS and induce the oxidative damage of DNA, proteins, and lipids (lipid peroxidation) [3]. However, like other antioxidants, under certain circumstances, flavonoids can act as prooxidants and promote the oxidation of other molecular species. Thus, flavonoids, including flavones, isoflavones, and flavanones, behave as antioxidants against peroxyl and hydroxyl radicals, whereas prooxidant activity is observed in the presence of Cu^2+^. Both the antioxidative and copper-initiated prooxidant activities of flavonoids are dependent on the number of hydroxyl substitutions in the flavonoid backbone. Mono- and dihydroxyflavonoids do not show visible prooxidative activity, while the presence of several hydroxyl groups, particularly in the B-ring, highly increases the production of hydroxyl radicals via Fenton chemistry. Prooxidative effects of flavonoids are possible in vivo if unbound ions of transition metals participate in oxidation processes. Flavonoids are able to reduce Cu(II) to Cu(I), thus enabling the generation of initiating radicals. In the presence of O_2_• or reducing agents such as flavonoids, Cu(II) can be reduced to Cu(I) with excess H_2_O_2_ to form HO• (Fenton-like reaction) [95]. As mentioned, Fe(II) catalyzes the formation of dangerous hydroxyl radical •OH in the presence of H_2_O_2_ [45]. It should also be noted that quercetin may form complexes with metal cations such as Mo(VI), Fe(II)/Fe(III), Cu(II), Zn(II), Al(III), Tb(III), Pb(II), and Co(II) [45,96]. The iron-binding properties of several polyphenolic compounds (quercetin, chrysin, 3-hydroxyflavone, 3′,4′-dihydroxy flavone, rutin, and flavone) were investigated by Liu and colleagues [97]. Surprisingly, quercetin, an oxygen-based ligand, bound Fe(II) stronger than ferrozine, the well-known strong nitrogen-based Fe(II)-chelator, and modulated cellular iron homeostasis under physiological conditions. In fact, many flavonoids, including quercetin, (+)catechin, rutin, and resveratrol can display either antioxidant or pro-oxidant activity depending on several parameters, such as applied dose and cellular conditions [98]. Thus, for example, resveratrol, which has been identified as a bioactive compound in Romanian propolis [99], exhibits prooxidant properties at high concentrations, by mobilizing endogenous copper ions and DNA-associated copper in cells. DNA-associated copper potentially may activate resveratrol and other phenolic compounds via a copper-redox reaction and induce the production of reactive oxygen and electrophilic intermediates, which may cause various lesions in DNA, including oxidative modifications of DNA bases, strand breaks, and adducts formation. Importantly, increased levels of copper have cytotoxic effects and could be considered an efficient mechanism for the induction of tumor cell death. Resveratrol may bind copper ions and DNA, forming a ternary DNA–resveratrol–Cu(II) complex, which induces DNA cleavage due to ROS generation through the Haber Weiss and Fenton reaction [100,101]). In the Fenton reaction, bound Cu(I) is oxidized to Cu(II), which immediately becomes available for binding to resveratrol and a new round of cycling. Approximately 20% of copper is located in the nucleus, in association with DNA bases, particularly guanine [102]. It seems that resveratrol is involved in the mobilization of guanine-associated copper, resulting in its redox recycling. Simultaneous generation of ROS ends in point mutations (deletions/substitutions) of guanine, whereas the addition of Cu(II) in the reaction mixture may induce changes in other bases as well. Enhanced accumulation of ROS may be the underlying mechanism of increased angiogenesis and metastasis, which together with point mutations, may accelerate the proliferation of tumor cells and shorten the survival time, at least in mice [98].

In addition to resveratrol, Cu^2+^ can be reduced to Cu^+^ in the presence of quercetin, which is usually found in propolis. In this reaction, a semiquinone radical (semi-oxidized quercetin) is formed. In a second electron transfer, this radical interacts with molecular oxygen and generates ortho-quinone and superoxide anion (O_2_^•^). Superoxide anion then reacts with catalytic Cu^+^ and generates H_2_O_2_, which is afterward converted into hydroxyl radical via the Fenton-type chemistry. Simultaneously, re-oxidation of Cu^+^ to Cu^2+^ further produces more ROS [46]. Thus, it is likely that propolis, i.e., its polyphenolic components, may induce DNA damage in the presence of transition metal ions. Flavonoids that are found in various propolis samples are powerful DNA-damaging agents. Based on this, it is suggested that flavonoids transfer electrons from transition metals to molecular oxygen, and produce superoxide anion and H_2_O_2_, which underlie pro-oxidant and cytotoxic effects on tumor cells. The pro-oxidant activities of polyphenols result in (i) cell cycle arrest; (ii) induction of apoptosis and DNA fragmentation; (iii) inhibition of proliferative signaling pathways, including epidermal growth factor receptor/MAPK (EGFR/MAPK), PI3K/Akt, and NF-κB; and (iv) anti-inflammatory effects [47,48,49,50].

### 3.2. Regulation of Cell Proliferation by Propolis and Its Polyphenolic/Flavonoid Compounds

Propolis and flavonoids are chemopreventive compounds that affect the proliferation, differentiation, and apoptosis of cancer cells, and their effect is especially pronounced in direct contact with cancer cells, for example, in the gastrointestinal system [3,7]. Antitumor properties of propolis and its phenolic constituents have been reported in various culture cell lines of human origin, including leukemia cells (HL-60, U937, MOLT-4, NALM-6, K562, CI41, HL-205, K-562-J, RS4;11, CEM, NB4, NALM-16, SUP-B15, and REH), colon cancer cells (SW480, HCT116), cervical cancer cells (ME180, Hela), glioblastoma cells (U87MG), lung carcinoma cells (A549), hepatocellular carcinoma cells (HepG2, SNU449), pancreatic cancer cells (PANC-1, BxPC-3), and mammary carcinoma cells (MCF-7) [103,104,105,106,107,108,109,110,111,112,113,114,115]. Direct cytotoxic effects of propolis and polyphenolic/flavonoid compounds were confirmed in mammary carcinoma cells [15,61] and primary culture of human papillary urothelial carcinoma cells [14,112]. We and others have shown that propolis and its flavonoids inhibit the proliferation of cancer cells by activating cytotoxic and apoptotic mechanisms [18,47,48,49,57,64,66,113,114,115,116,117,118]. It seems that the cytotoxicity of flavonoids depends on their chemical structure, concentration, and type of leukemic cells. Reynal et al. [119] found that genistein induces a time- and dose-dependent effect on both myeloid and lymphoid leukemic cell lines. Plasma concentrations of genistein (1–20 µM) after a soy-enriched diet results in a loss of clonogenicity in leukemic cells. In leukemic mice fed on a diet enriched with 0.5% genistein, survival time was significantly increased in comparison with mice on a normal diet. The moderate antileukemic effect of genistein in vivo is probably due to its very rapid inactivation (15 min) in mice. In another study, genistein did not exceed 1 µM concentration in the blood of mice fed with a 0.6% soy extract supplement [120]. The antitumor activity of curcumin, epigallocatechin gallate (EGCG), or indole-3-carbinol (I3C) in MDA-MB-231 breast cancer cells was confirmed through the inhibition of clonogenic growth by 55% to 60%, an increase in basal caspase-3/7 activity by 1.5 to 2 times, and a prolonged doubling time, while genistein did not show an anticancer effect on the same cells [121]. Similarly, animal studies did not show the inhibition of tumor growth in the MDA-MB-231 cell xenograft with the ~1 μM concentration of genistein in serum [122]. This probably indicates the lack of a protective effect of genistein in estrogen receptor-α-negative tumors [123].

CAPE inhibited the proliferation of the colorectal cell line SW480 by downregulating the expression of β-catenin, c-myc, and cyclin D1 [124]. The dose-dependent effect of CAPE on the proliferation of LNCaP, DU-145, and PC-3 human prostate cancer cells was confirmed in a xenograft model using LNCaP cells and nude mice [125]. In addition, propolis and CAPE in a dose-dependent manner inhibited self-renewal of cancer stem cells, progenitor formation, and clonal growth.

#### Key Mechanisms of Propolis and Its Polyphenolic/Flavonoid Compounds Involved in Inhibition of Cell Proliferation

Several mechanisms have been implicated in the inhibition of cell proliferation: (i) inhibition of the activity of tyrosine-specific protein kinases [126,127,128], such as inhibition of PKC, which is one of the key enzymes in the regulation of cellular proliferation and tumor growth; (ii) induction of the differentiation of carcinoma cells [3,13,126,127,128,129]; (iii) transcriptional changes in cell cycle- and apoptosis-related genes such as NF-κB, Bcl-X(L) and COX-2; (iv) binding to the estrogen receptor and inhibition of an estrogen receptor-positive human breast cancer cells [130,131]; (v) induction of apoptosis, (vi) downregulation of the expression of mutated H-Ras and p53 tumor suppression gene [3,13,126], (vii) modulation of gene methylation and re-expression of tumor suppressors or other genes silenced by aberrant DNA methylation [119,130,131], (viii) inhibition of pro-oxidative enzymes xanthine oxidase, cyclooxygenases, or lipooxygenases [3,13,126,127], and (ix) some flavonoids are a direct poison for topoisomerase II (TopoII), through the stabilization of double strand breaks in the TopoII-DNA cleavage [4,9,10,11,12,13].

Flavonoids exhibit a wide range of activities that orchestrate signal transduction. They downregulate growth factors (epidermal growth factor (EGF) and vascular endothelial growth factor (VEGF)), modulate survival signaling pathways (ERK, JNK, AP-1, and NF-κB), and reduce the expression of cell cycle regulators (cyclinD1, cdk4/6, p21, and p53) and apoptosis regulators (PARP, ceramides, and caspases) [1,2,3,13,47,48,49,126].

Cell cycle progression is a sequential and highly regulated process that drives the division of mammalian cells through the G1, S, G2, and M phases. G1-S or G2-M transitions are regulated by checkpoints that may arrest cell cycle progression in environmental stress conditions. As balanced interactions between cyclins, cyclin-dependent kinases (CDK), and CDK inhibitors (CDI) regulate progression through the cell cycle [132], altered expression of the cell-cycle-specific proteins by dietary components potentially may sustain the proliferation of neoplastic cells and serve as “effect” biomarkers of their cytotoxic activity. The phenolic compounds of propolis, such as genistein, quercetin, kaempferol, myricetin, luteolin, chrysin, and apigenin, have been confirmed to modulate cell proliferation through an effect on the cell cycle by inducing CDI (p21 and p27) and inhibiting CDK4, CDK2, cyclin D1, and cyclin E [133,134,135,136,137,138,139]. Kabała-Dzik et al. [49] compared caffeic acid (CA) and CAPE activity on triple-negative human Caucasian breast adenocarcinoma cell line (MDA-MB-231). They showed that CAPE displays higher cytotoxic and apoptotic activity compared to CA, and that CAPE induces cell cycle arrest in the S phase (time and dose-dependently), while CA demonstrated this effect only at 50 and 100 μM concentrations. Algerian propolis and its combination with doxorubicin decreased cell viability, prevented cell proliferation and cell cycle progression, induced apoptosis by activating caspase-3 and -9 activities, and increased the accumulation of chemotherapeutic drugs in MDA-MB-231 cells by blocking P-gp function and cell cycle arrest in the S phase [136]. Jiang and co-authors also demonstrated that Chinese propolis selectively inhibits the proliferation of human gastric cancer SGC-7901 cells by triggering both death receptor- and mitochondria-induced apoptosis, and cell cycle arrest in the S phase [137]. In the study by Zang et al. [138], it was shown that flavones (luteolin, chrysin, and apigenin) and flavonols (quercetin, kaempferol, and myricetin) can induce cytotoxicity to OE33 cells (a human esophageal adenocarcinoma cell line) by causing G_2_/M arrest and induction of apoptosis. At low μM doses, polyphenols activated hormetic signaling pathways involving various kinases and transcription factors [138]. These pathways, in turn, activate the expression of genes encoding stress resistance proteins, such as antioxidant and detoxifying enzymes, protein chaperones, neurotrophic factors, and other cytoprotective proteins. Of note, one such pathway is the Nrf2/ARE pathway [139,140,141]. At low concentrations, polyphenolic compounds are not cytotoxic, but cytostatic. They usually arrest the cell cycle in the S/G2 and mitotic phases, leading to cell death. In addition, they act on multiple kinases implicated in cancer pathogenesis [142].

Propolis, as well as flavonoids derived from propolis, such as galangin, is a potent COX-2 inhibitor [1,2,3,13,47,48,49,126,143]. Flavonoids and caffeic acid analogs are also effective xanthine oxidase inhibitors [1,2,3,13,47,48,49,126,143]. COX-2 and xanthine oxidase are ROS-producing pro-oxidant enzymes that contribute to inflammation. There is considerable evidence suggesting that angiogenesis and chronic inflammation are mutually interdependent. Inhibition of angiogenesis has an anti-inflammatory effect. For example, EEP and components of propolis, such as CAPE, quercetin, resveratrol, and genistein, exhibit anti-inflammatory and anti-angiogenic properties in urothelial cancer [1,2,3,13,47,48,49,126,143].

Some flavonoids from propolis, such as galangin, genistein, baicalein, hesperetin, naringenin, and quercetin, suppressed the proliferation of an estrogen receptor (ER)-positive human breast cancer cell line MCF-7 [1,2,3,13,144,145,146]. Flavonoids possess a binding affinity for ER-α estrogen receptors expressed in the breast, endometrium, and ovaries, and for ER-β estrogen receptors in the brain, blood vessels, lungs, and bones. CAPE greatly contributes to the estrogenic activity of propolis and shows a higher affinity for ER-β than to ER-α. Studies have shown that CAPE is a selective agonist of the ER-β. As ER-β does not contribute to the estrogenic effect on the ER-positive cancer cells, it is assumed that CAPE acts as a modulator of the estrogen receptor [146]. In addition, chrysin, yet another active compound from propolis, blocks the conversion of androgens into estrogens by inhibiting aromatase, hence increasing testosterone levels. Besides chrysin, galangin, as well as flavones such as naringenin, deplete estrogen synthesis via aromatase inhibition. Flavonoids and flavonoid derivatives possess two structural features that contribute to the aromatase inhibitory activity. Firstly, the A and C rings of the flavonoids are structurally similar to the D and C rings of the aromatase substrate androstenedione, and secondly, the presence of oxo-group at the C4 position, which is essential for binding iron atoms of the heme group of aromatases [147]. Similar activity was also observed for apigenin [148,149,150,151]. In breast carcinoma cells, the antiproliferative effects of genistein were mediated by an intracellular metabolite, which inhibited cell cycle progression. In addition, genistein may affect apoptosis, angiogenesis, and metastatic processes. Various molecular effects modulated by genistein include the downregulation of growth factors (EGF and VEGF), and modifications of survival signaling pathways (ERK, JNK, AP-1, and NF-κB), cell cycle regulators (cyclinD1, cdk4/6, p21, and p53), and regulators of apoptosis (PARP, ceramides, and caspases) [152,153,154].

Regarding propolis, it has been shown that it inhibits cell cycle progression by suppressing expressions of cyclin A, cyclin B, and Cdk2 and by stopping proliferation at the G2 phase, by increasing levels of p21 and p27 proteins, and through the inhibition of telomerase reverse transcriptase (hTERT), leading to telomere shortening and cancer cell death [1,2,3,4,9,10,11,12,13,155,156].

### 3.3. Epigenetic Regulation by Propolis and Its Polyphenolic/Flavonoid Compounds

Growing evidence suggests that flavonoids may affect tumor progression by acting at chromatin remodeling and interfering with epigenetic alterations. Chromatin remodeling refers to chemical modifications of DNA and histones, including DNA methylation and multiple histone modifications, such as acetylation, phosphorylation, sumoylation, and ubiquitination. Post-translational modifications of histones are important for the epigenetic regulation of gene expression. In that regard, two important modifications of histones are processes of acetylation and deacetylation. Histone acetylation is catalyzed by the histone acetyltransferases (HATs), whereas 18 different histone deacetylases (HDACs) catalyze histone deacetylation. HDACs are divided into four classes based on their structural similarity [13,157,158], and are distributed in the cytoplasm, mitochondria, and nucleus. Seven members of HDACs are known as sirtuins (SIRTs—SIRT1—SIRT7) [157,158]. Noncoding RNAs, such as microRNAs (miRNAs) and short interfering RNAs (siRNAs), as well as some other epigenome constituents, are also implicated in the control of epigenetic mechanisms that regulate gene expression. The genetic mutations along with the epigenetic alterations can be found in cancer cells. Several dietary and nutritional compounds have been reported to affect epigenetic modifications and gene expression of tumor suppressors and other cancer-related genes. The epigenetic alterations are reversible, and under certain conditions may be induced by natural polyphenols [157]. It is known that enzymes regulating epigenetic processes, DNA methyltransferases (DNMTs) and HDACs, are aberrantly upregulated in both developed cancer and in early phases of carcinogenesis [158].

The network of SIRT1-modulated signals is very complex and involves direct interactions of SIRT1 with proteins involved in cell survival, DNA repair, and cell cycle/apoptosis [157,158,159,160,161]. If the tight homeostatic balance between the acetylation and deacetylation changes due to the abnormal activities of either HATs or HDACs, this will end in pathological situations, as seen in cancer [158]. Not surprisingly, HDAC inhibitors have been in the focus as therapeutic options in cancer chemotherapy. On the other hand, dietary polyphenols from propolis may act as HDAC inhibitors, which might be of great importance in the prevention and therapy of cancer and other diseases [157,158,159,160,161,162,163,164,165,166].

It has been shown that specific enzymes and methylated DNA binding proteins may suppress the expression of tumor suppressor genes, leading to tumor formation and progression. Genes participating in cell cycle regulation, DNA repair, angiogenesis, and apoptosis are inactivated by hypermethylation of their respective 5′CpG islands. Some key regulatory genes influenced by DNA hypermethylation and histone acetylation and deacetylation are genes coding E-cadherin, pi-class glutathione S-transferase, the tumor suppressor cyclin-dependent inhibitor 2A (CDKN2A), PTEN, and insulin-like growth factor (IGF-II), among others. Accordingly, there are growing efforts to discover and develop compounds capable to act as DNA demethylating agents and histone deacetylases inhibitors (HDACi) [162]. One potential approach to prevent hypermethylation of DNA and inactivation of tumor suppressor genes is to use natural polyphenols from propolis, such as genistein, several isothiocyanates, catechin, lycopene, and quercetin.

#### 3.3.1. Propolis and Its Flavonoids as Inhibitors of Methyltransferase and Histone Deacetylase

Several studies have shown that compounds from propolis may target proteins participating in the epigenetic regulation of gene expression. Raynal et al. [163] and Fang et al. [164] have shown that genistein may reactivate the expression of tumor suppressor genes repressed by aberrant DNA methylation. In particular, genistein (1–20 µM) induced a re-expression of tumor suppressor genes coding for cyclin-dependent kinase inhibitors, *p57*^KIP2^ in human leukemia cell lines HL-60, MOLT-3, Raji, KG1a, and *p15*^CDKN2B^ in mouse cell line L1210 [163].

CAPE is an epigenetic modulating agent that affects the activities of oncogenes and tumor suppressor genes. Treatment with CAPE and propolis resulted in an accumulation of acetylated histone proteins in MCF-7 (ER+) and MDA-MB-231 (ER-/PR-/Her2-) cells with concomitant downregulation of ERs and progesterone receptors (PRs) in MCF-7 cells, and increased expression of ERs and downregulation of EGFR in MDA-231 cells [165]. The re-expression of ER-α in triple-negative breast cancer cells following treatment with CAPE might indicate that CAPE could make triple-negative breast cancer patients susceptible to chemoprevention and anti-estrogen therapy if used in combination with hormone-based therapy, in either the adjuvant or metastatic setting. In addition, these products decreased the expression of the p-Her2 protein in SKBR3 (Her2+) cells. Interestingly, propolis, when normalized for CAPE content, is more potent than CAPE alone. This activity may be explained by the presence of caffeic acid, the hydrolyzed product of CAPE, which is present together with CAPE in propolis, and probably enhances the HDAC inhibitory effect of CAPE. CAPE has the ability to reduce cyclin D1 expression by sevenfold and may inhibit histone deacetylases directly or indirectly. Moreover, in MCF-7 and MDA-MB-231 breast cancer cells, other propolis components, such as caffeic or chlorogenic acid, partially inhibited the methylation of the promoter region of the retinoic acid receptor beta (*RARB*) gene, suggesting that natural compounds from propolis may function as epigenetic modulators. Assumpção et al. [166] indicated that some chemicals present in the EEP have DNMTi activity and can reverse the epigenetic repression of the tumor suppressor Ras association domain family member 1 (RASSF1A), in the BT-549 triple-negative breast cancer cell line. Two isoforms of this gene, *RASSF1A* and *RASSF1C*, contribute to cancer development and progression. CpG island of the *RASSF1A* isoform is usually hypermethylated in various cancers and may be viewed as a marker for early cancer detection and prognosis, while the CpG island of *RASSF1C* remains unmethylated. According to Morel et al. [167], epigenetic drugs targeting DNA methyltransferases are a promising approach for the improvement of cancer therapy, as the combined use of DNMTi at low doses might reverse resistance to cytotoxic agents, in part by abolishing the acquired epigenetic alterations associated with the therapy resistance.

CAPE also reversed UV-induced epigenetic modifications in human dermal fibroblasts via direct inhibition of several HATs, including p300, CREP-binding protein (CBP), and p300/CBP-associated factor (PCAF) [168].

Flavonol quercetin is also an epigenetic modulator. Hypermethylation of the tumor suppressor gene *p16INK4a* (p16, cyclin-dependent kinase inhibitor 2A) was reversed by quercetin after 120 h of treatment in the human colon cancer cell line RKO [169]. Quercetin, as well as other catechol-containing polyphenols, may indirectly inhibit the activity of DNMTs and DNA methylation by modifying the intracellular concentrations of S-adenosyl methionine (SAM) and S-adenosyl-L-homocysteine (SAH). SAM is the methyl donor for most methylation reactions, whereas SAH is a potent inhibitor of all methyltransferases. Ma et al. [170] studied the methylation of genes involved in cell cycle control (*p16INK4a* or *CDKN2A* and RASSF1A), and estrogenic transduction (*ER-β* or *ESR2*). Hypermethylation of these genes was confirmed in T24 cancer cells, which agrees with other reports on various human cancers [169,170,171,172,173,174,175,176,177]. Demethylation of these genes by quercetin restores their transcriptional activity, indicating that p16, RASSF1A, and ER-β are involved in cell growth arrest and apoptosis in T24 cells after quercetin treatment. Moreover, the DNA methylation levels of ER-β, p16INK4a, and RASSF1A were strongly reduced (from 35% to 70%) in the presence of quercetin. When quercetin was combined with trichostatin A (TSA, HDAC inhibitor), they cooperatively induced the death of human leukemia cells [177]. In anti-human esophageal cancer 9706 cells (Eca9706 cells), Zheng et al. [169] demonstrated the chemopreventive effect of nanoliposomal quercetin combined with butyrate. Epigenetic modifications targeted both DNA methylation and histone acetylation, and quercetin and butyrate acted together as HDAC inhibitors via HDAC-NF-κB-signaling. Expression of E-cadherin was also increased, suggesting that the reversal of epithelial–mesenchymal transition (EMT) may contribute to the inhibition of migration/invasive potency of Eca9706 cells, either via HDAC inhibition or through the quercetin and butyrate-mediated inactivation of NF-κBp65. It is well known that flavonoids from fruits, vegetables, etc., can suppress the proinflammatory signaling pathways (such as NF-κB), and thus may help in the prevention and even therapy of cancer [172,173]. Quercetin also decreased the acetylation of histone H3 and expression of survivin in human prostate cancer cells, by inhibiting the binding of transcription factor Sp1 to the *survivin* promoter [174]. The H3 deacetylation could be involved in the inhibition of survivin expression and the later sensitization to TRAIL-induced apoptosis [173]. The detailed analysis of the mechanisms underlying this effect reveals that the increase in cell death was mediated by enhanced activation of the caspase cascade, together with the downregulation of survivin in an ERK- mitogen- and stress-activated kinase 1 (MSK1) dependent pathway. According to the results of Kim et al. [173], quercetin is capable to activate HAT via ERK and JNK signaling pathways. In addition, quercetin demonstrated a weak inhibitory effect on HDAC activity in HL-60 cells. It induced FasL-related apoptosis by increasing FasL expression via transcriptional activation of the *FasL* gene. Quercetin-mediated upregulation of FasL was due to the increased accumulation of c-Jun and AP-1 activation and promotion of histone H3 acetylation, at least partially via ERK and JNK signaling pathways [174].

Quercetin also blocked the binding of different trans-activators, including p300, CREB2, c-Jun, C/EBPβ, and NF-κB, to the promoter of the proinflammatory gene *COX-2* [164]. Consequently, quercetin reduced levels of the COX-2 protein. Generally, a COX-2 decrease is considered beneficial for successful cancer chemoprevention [174,175,176,177]. Furthermore, quercetin may induce significant histone hyperacetylation at 75 and 100 μM concentrations in human leukemia cells, suggesting the possible role of histone hyperacetylation in the anticancer activity of quercetin in vitro [178,179]. In conclusion, numerous studies have shown that quercetin may affect the activation of different signaling pathways and chromatin remodeling, and act as a chemopreventive agent [1,2,3,4,13,177,178,179,180,181,182].

Some of the proposed mechanisms of epigenetic regulation by different polyphenolic antioxidants were summarized by Malireddy et al. [178]. They suggest that flavonoids may act either as (i) antioxidants, attenuating oxidative stress; and/or as (ii) direct modulators of DNMT, HDACs, and HATs. In addition, polyphenols behave as pro-oxidants in the presence of transition metals and oxygen, or act in concert with oxidases, and induce oxidative stress, which may modulate the cellular epigenetic fingerprint via oxidant and thiol-redox signaling. It also seems possible that metabolites formed by biotransformation of flavonoids by the cellular phase-I and phase-II xenobiotic-metabolizing enzymes regulate the cellular epigenome via signaling cascades. According to Malireddy et al. [178], for now, it is unclear whether the epigenetic regulation by redox-active phytochemicals is dependent either upon the oxidants produced by the prooxidant actions or the antioxidant nature of polyphenol antioxidants.

Huang et al. [179] investigated Taiwanese green propolis (TGP) extract that contains a variety of chemical components, including Propolin G, an active anticancer compound. Propolin G was used to develop an anticancer agent NBM-HD-3, a histone deacetylase inhibitor (HDACi) that exerts anticancer activity by modulating PTEN and AKT in brain cancer cells.

#### 3.3.2. Propolis and Its Flavonoids Modulate miRNAs

Some dietary bioactive compounds, including flavonoids, are known to reduce the tumor burden either by directly modulating miRNAs or through indirect action on their target genes [183,184,185,186]. MicroRNAs (miRNAs) are small regulatory molecules, 20–25 nucleotides in length that modulate protein expression at the post-transcriptional level, by binding to the 3′-untranslated region (3′-UTR) of target mRNAs. This results in mRNA destabilization and degradation [186,187,188,189,190]. Various cellular processes, such as cell differentiation, maintenance of homeostasis, and immune responses, are thought to be controlled by miRNAs, and the altered expression of miRNA is associated with most human malignancies [186,187,188,189]. Thus, miR-203, located at chromosome 14q32-33, is abundantly expressed in the skin, and plays an important role in epidermal development, homeostasis of human keratinocytes, and hair follicle morphogenesis [189]. It is known that EGCG offers protection against UV-B-induced damage in normal human dermal fibroblast (NHDF) by regulating specific miRNAs [189]. Even though a significant number of studies have identified flavonoids as regulators of miRNAs in various malignancies, the direct regulatory effect of flavonoids on miR-203 is not completely clarified in skin cancers [184,185,186,187,188,189,190]. It has been shown that curcumin may induce epigenetic changes and upregulate miR-203 in human bladder cancer cells, along with decreasing disease progression [181]. Dietary intake of 4% curcumin significantly altered the miRNA expression profile in murine melanoma, suggesting a probable mechanistic link between the skin-specific miR-203 and curcumin that needs to be investigated further [189,190]. Several lines of evidence indicate that curcumin exerts its therapeutic effects by regulating miRNA expression (e.g., miR-1, miR-7, miR-9, miR-19, miR-21, miR-34a, and miR-181), probably modifying cellular and molecular pathways underlying cancer pathogenesis [190]. Curcumin, EGCG, quercetin, genistein, pterostilbene, resveratrol, capsaicin, ellagic acid, benzyl isothiocyanate, phenethyl isothiocyanate, sulforaphane, indole-3-carbinol, 3,30-diindolylmethane, diallyl disulfide, betulinic acid, and oleanolic acid, have all demonstrated microRNA regulatory activities in various cancers, including the regulation of the malignant mesothelioma microRNAs [189].

Several detailed and informative reviews on the association between miRNA and cancer have been published previously and are beyond the scope of this review [186,187,188,189,190].

### 3.4. Regulation of Cell Death by Propolis and Its Polyphenolic/Flavonoid Compounds

**Figure 1:**
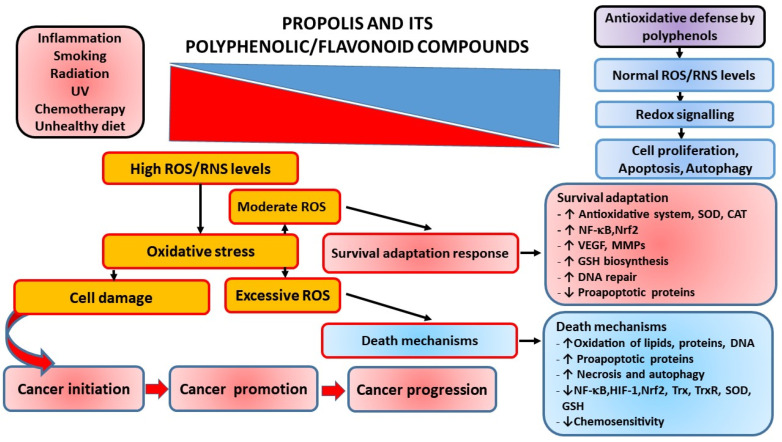
Interrelationship between ROS, oxidative stress, and inflammation-induced immune dysregulation and cancer pathophysiology. A number of factors induce the generation of high levels of reactive radicals (ROS), leading to oxidative stress (OS) and OS-induced inflammation. ROS can activate transcription factors NF-κB, AP-1, and HIF-1α that promote expression of pro-inflammatory genes, and activation of these redox-sensitive pathways may result in various cellular responses. ROS can serve as signaling molecules maintaining physiological functions such as cell growth, proliferation, and survival. Excessive production of ROS leads to oxidative damage of cellular macromolecules and cell death, while a moderate level of ROS leads to cell adaptation and survival through increased synthesis of antioxidant molecules, induction of angiogenesis factors, and increased DNA repair processes. In addition, ROS can stimulate signal transduction pathways and activate the key transcription factors such as Nrf2 and NF-kB. The altered patterns of gene expression induced by excess ROS contribute to the carcinogenesis process. Chronic inflammation upregulates cellular levels of inflammatory mediators, including COX-2, reactive oxygen and nitrogen species, and inflammatory cytokines, and activates protooncogenes. Depending on the overall functions and ratio of inflammatory mediators, the inflammatory response may be pro- or anti-tumorigenic. Finally, the antioxidant regulatory mechanisms that modulate and balance host defense, inflammation, and OS are indicated. In general, the strategies of antioxidant and anti-inflammation therapy as a defensive approach against cancer include: (1) use of antioxidants, (2) suppression of inflammation, and (3) stimulation of repair processes. Note: ROS, Reactive oxygen species; NO, Nitric oxide; NF-κB, Nuclear factor kappa-light-chain-enhancer of activated B cells; UV, ultraviolet radiation; SOD, Superoxide dismutase; CAT, Catalase; Nrf2, nuclear factor E2-related factor 2; VEGF, Vascular endothelial growth factor; MMPs, Matrix metalloproteinases; GSH, Reduced glutathione; HIF-1, Hypoxia-inducible factor-1; Trx, Thioredoxin; TrxR, thioredoxin reductase.

The growth rate of pre-neoplastic or neoplastic cells is higher than that of normal cells due to disturbed functioning of their cell-growth and cell-death mechanisms. Many studies have revealed that apoptosis- and/or autophagy-related signaling pathways are modulated by propolis and polyphenols. The complex network of functional interconnections between apoptosis and autophagy in polyphenol-mediated cancer therapy may be the key factor involved in the actions of polyphenols (Figure 1).

#### 3.4.1. Modulation of Apoptosis Pathway by Propolis and Its Polyphenolic/Flavonoid Compounds

Apoptosis is one of the most important mechanisms of defense against cancer as it removes potentially dangerous, mutated cells. Apoptotic death is required for various physiological processes, such as the maintenance of cellular homeostasis and development. Chemopreventive compounds, including propolis, phenylethyl isothiocyanate, sulforaphane, EGCG, curcumin, apigenin, quercetin, and resveratrol, have been confirmed to induce apoptosis [1,2,3,4,13,46,47,48,191,192,193,194,195]. Moreover, experimental data indicate a direct relationship between glutathione levels in tumor cells, cytotoxicity, and apoptotic death [13,75,129].

It has been pointed out that the concentration of flavonoids and the level of free radicals are the key factors that determine proliferation and/or cell death by apoptosis and/or necrosis. Thus, Ramos [193] proposed that quercetin at low concentrations can activate MAPK pathways, which, in turn, increases the expression of survival genes (c-fos and c-jun) and defense genes (glutathione-s-transferase), while quercetin at high concentrations activates the caspase cascade that leads to apoptosis. Flavonoids can interfere with metabolic cellular processes and induce different effects under the same conditions. Ferraresi et al. [196] showed that short-term treatment with quercetin exerts antioxidative and antiapoptotic effects, whereas chronic treatment results in prooxidative and proapoptotic activities of quercetin. According to the authors, a depleted level of reduced glutathione (GSH) is associated with apoptosis, indicating that the GSH amount largely determines the pro- or antioxidant mode of quercetin action [196]. Interestingly, flavonoids trigger apoptosis only in transformed cells, and their sensitivity to antitumor substances depends on their ability to synthesize GSH in response to oxidative stress [13,46,63,197]. Moreover, the majority of these chemopreventive compounds trigger mitochondria-mediated apoptosis due to the pro-oxidative action on transformed cells (Figure 1). The resulting ROS-induced mitochondrial membrane permeabilization (MMP, e.g., loss of mitochondrial intrinsic transmembrane potential), mitochondrial cytochrome c release, and/or mitochondrial swelling occurs mainly through the mitochondrial permeability transition (MPT), and/or possibly via the redox-regulated activity of the pro-apoptotic members of the Bcl-2 family (i.e., Bax and Bak) (reviewed in [46,129]).

Thus, potential biomarkers of the cytotoxic effect of propolis and its flavonoids may be estimated by monitoring their effects on mitochondrial caspases and other apoptosis-related proteins. In various types of cells, quercetin-mediated apoptosis was related to stress proteins, microtubule disruption, activation of NF-κB pathway, COX-2, p53, survivin, c-Jun NH2-terminal kinase (JNK), mitogen-activated protein kinase kinase-extracellular signal-regulated kinase (MAPKK-ERK), Bcl-2 family proteins, heat shock proteins, DNA topoisomerase II, cytochrome c release, and caspase activation [129,173,174,198]. Quercetin also triggered the extrinsic apoptosis pathway by activating caspase-8 and inducing Bid cleavage, conformational changes in Bax, and cytochrome *c* release in human leukemia HL-50 cells [173].

Tumor necrosis factor-related apoptosis-inducing ligand (TRAIL) is a member of the necrosis factor (TNF) superfamily that also includes TNF, Fas ligand, lymphotoxin, CD27L, OX40, CD30L, and CD40L [199]. TRAIL is the most potent anticancer agent in the TNF superfamily based on its selective cytotoxicity in transformed cells. Quercetin sensitized TRAIL-induced cytotoxicity in non-small cell lung cancer (NSCLC) cells through two independent pathways: induction of death receptor 5 (DR5) and inhibition of survivin expression. These pathways probably represent the underlying mechanism of the preventive activity of quercetin in lung cancer. Hence, the potentiation of TRAIL-induced NSCLC cell death could be considered a potential target in lung cancer therapy and prevention [199]. Quercetin can also sensitize the acute myeloid KG-1 cells against TRAIL. Perhaps the combined therapy with these agents might improve the clinical efficacy of TRAIL in patients with acute myeloid leukemia (AML) [200].

The extrinsic pathway of apoptosis is also triggered by propolis [200,201,202,203,204,205]. Thus, Szliszka et al. [201] have shown that EEP (50 μg/mL) markedly increased the apoptosis-inducing potential of TRAIL in the HeLa cell line. TRAIL, as a member of the TNF superfamily, initiates programmed death in cancer cells by interacting with the death-domain-containing receptor TRAIL-R1 (death receptor 4-DR4) and/or TRAIL-R2 (death receptor 5-DR5) [201].

Similarly, ethanol extract of Brazilian green propolis sensitized prostate cancer cells to TRAIL-induced death, upregulated the expression of TRAIL-R2, and reduced the activity of NF-κB [202]. Co-treatment of TRAIL with artepillin C, a major ingredient of Brazilian propolis, induced activation of caspase-8 and caspase-3, and disrupted the mitochondrial membrane potential [203]. CAPE, yet another compound present in propolis, induced cell cycle arrest and apoptosis, and reduced the expression of NF-κB in MDA-MB-231 and MCF-7 human breast cancer cells [204]. In PC3 prostate cancer cells, CAPE triggered apoptosis in a dose-dependent manner. This pro-apoptotic effect was accompanied by the loss of expression of apoptosis inhibitors: cIAP-1, cIAP-2, and XIAP [205]. Cavaliere et al. [206] demonstrated that CAPE induces apoptosis via the mitochondrial intrinsic pathway in lymphoblastoid cell line PL104.

The antitumor effect of the water-soluble derivatives of propolis (WSDP) from Croatia and Brazil has been studied by Orsolic and Basic in mammary carcinoma cells (MCA), human epithelial carcinoma cell line (HeLa), and Chinese hamster lung fibroblast cells (V79) [21]. The proportion of apoptotic MCA cells increased from 20% (in control group) to 24% and 26% following treatment with 50 μg/mL of Brazilian and Croatian propolis, respectively. In control HeLa cells, there were 2% apoptotic cells. The number of apoptotic cells was increased to 10% with Croatian propolis and 9.5% with Brazilian propolis. On the contrary, the percentage of apoptotic V79 cells was reduced following treatment with Brazilian and Croatian propolis. These results also indicate different sensitivity of cancer cells and normal fibroblasts to propolis. Propolis and its chemical constituents artepillin C, baccharin, drupanin, CAPE, and chrysin, demonstrate powerful cytotoxic effects and induce apoptotic death in breast, cervical, colon, intestine, liver, lung, prostate, skin cancers, and leukemia, in vitro and in various animal models [7,8,9,10,11,12,13].

In a rat model, dietary curcumin enhanced the apoptotic index in azoxymethane-induced colonic tumors [207,208,209,210]. Azoxymethane treatment in rats induces the formation of aberrant crypt foci, but their number was significantly reduced with resveratrol (200 µg/kg per day for 100 d), together with the decreased level of Bax and upregulation of p21 in the crypts [211]. Administration of resveratrol (25 mg/kg) for 3 weeks increased the apoptotic index and decreased angiogenesis in a xenograft model with MDA-MB231 cells [212]. Similarly, resveratrol was confirmed as a bioactive compound of Western Romanian propolis at a dose of 8 mg/kg body weight [99,213]. Conversely, when B16M tumor cells were inoculated into mice, 20 mg/kg resveratrol did not exert any effect on tumor growth [214]. The poor bioavailability of resveratrol in tumor therapy can be increased by nanoparticles. Nanotechnology approaches have been broadly utilized to obtain better solubility, enhance oral bioavailability, increase stability, and regulate the release of resveratrol. The resveratrol nanoparticles have markedly improved anticancer properties of resveratrol both in vitro and in vivo, indicating this approach as a potential strategy against various cancers [98,215,216,217]. González-Sarrías et al. [217] showed that milk-derived exosomes (EXO) as nanocarriers can increase the bioavailability and anticancer activity of curcumin and resveratrol in breast tissue by increasing their antiproliferative activity, induction of apoptosis, cell cycle alteration, and caspase activation, even at nanomolar concentrations. The application of EXO-curcumin and EXO-resveratrol and their internalization via clathrin-mediated endocytosis inhibits the mechanism of chemoresistance mediated by the ABC transporter and increases the anti-cancer effect. Flavonoids induce apoptotic death in many cancer cells including a variety of leukemic cell lines, but sparing the normal cells [3,13,46,218,219]. They achieve apoptotic effects via several mechanisms: (i) inhibition of DNA topoisomerase I/II activity; topoisomerases play an essential role in maintaining DNA topology during replication, transcription, and recombination [3,13,46,63,185,220,221], and flavonoids, such as quercetin, acacetin, apigenin, kaempferol, morin, and luteolin, inhibit topoisomerase I-catalyzed relegation of the DNA backbone [46,66,181,220,221]; (ii) suppression of ROS production; free radicals such as superoxide anion and hydroxyl radical and non-free radicals such as hydrogen peroxide and hypochlorous acid may induce oxidative damage of proteins, DNA, and RNA, as well as oxidation of fatty acids in plasma membranes, which further can enhance the risk of mutations and encourage carcinogenesis [1,2,3,4,9,10,11,12,13,14,15,16,17,18,19,20,46,47,48,49,50,63,181,220,221]; (iii) modulation of expression of heat shock proteins [3,13,14,170,181,222], (iv) regulation of signaling pathways [34,51,60,100,213] and cytochrome c release, leading to the activation of caspase-9 and -3 [1,2,3,4,9,10,11,12,13,14,15,16,17,18,19,20,46,47,48,49,50], reduced expression of Bcl-2 and Bcl-X(L) and overexpression of Bax and Bak, stimulation of NF-κB activity, activation of endonucleases, and downregulation of Mcl-1 protein [1,2,3,4,9,10,11,12,13,14,15,16,17,18,19,20,46,47,48,49,50]; and (v) one of the mechanisms involved in propolis-induced apoptosis may be related to telomerase activity inhibition [44,64,65,66,218,223].

#### 3.4.2. Propolis and Its Polyphenolic/Flavonoid Compounds Reduce Expression of Autophagy-Related Proteins in Cancer

Autophagy, a mechanism of cell death that involves the degradation of cytoplasmic material, damaged organelles, and aggregation-prone proteins in lysosomes, is known to play a dual role in tumors [219,224]. Macroautophagy (herein referred to as autophagy) is regulated by the highly conserved autophagy-related genes (*Atg* genes) and ubiquitin-like conjugation systems (Atg12 and LC3 systems), which act as regulators of autophagosome formation. In particular, the Atg5-Atg12-Atg16 and lipidated Atg8-phosphatidyl ethanolamine (mammalian homolog is LC3) conjugation systems regulate nucleation, expansion, and maturation of autophagosome, and ultimately fusion with the lysosome. In the early stages of tumor growth, autophagy acts as a tumor suppression mechanism, based on its role in the clearance of the scaffold protein p62/SQSTM1 and damaged organelles, and prevention of oxidative stress and genomic instability. These processes inhibit the malignant transformation of normal cells into tumor cells. In the later stages of the tumor, autophagy functions as the survival mechanism of the cancer cells. Under stress conditions, which may include a deficiency in nutritional components (amino acids or glucose) and hypoxia that often occurs in solid tumors, as well as during tumor response to therapy, tumor cells can utilize autophagy to survive. In fact, during stress conditions, autophagy promotes the survival of tumor cells, metastasis formation, and resistance to chemotherapy, suggesting that the inhibition of autophagy may enhance tumor cell killing following chemotherapy. Indeed, the inhibition of autophagy by silencing *Atg* genes or using chloroquine to inhibit autolysosomal degradation suppressed pancreatic tumor growth by inducing accumulation of ROS, DNA damage, and impairment of mitochondrial functions. More importantly, autophagy inhibition led to significant tumor regression and prolonged survival of mice carrying pancreatic cancer xenografts. Moreover, for chloroquine, an autophagy-related effect on tumor regression was demonstrated for propolis and its polyphenolic components, including CAPE, artepillin C, kaempferol, genistein, quercetin, galangin, luteolin, silibin, and rottlerin [7,115,225,226,227,228,229,230]. These polyphenols may trigger cell death by modifying different autophagy-related mechanisms. Thus, artepillin C showed high autophagy-inducing activity, as evidenced by the prominent upregulation of LC3-II in prostate cancer CWR22Rv1 cells, while galangin increased LC3-II expression in laryngeal cancer cells [225]. Similarly, ethanol-extracted Chinese propolis and CAPE increased the expression of LC3-II and degradation of p62, thus inducing autophagy in inflammation-stimulated breast cancer cells MDA-MB-231 [7,115]. In human hepatic cancer cells SK-HEP-1, kaempferol increased protein levels of LC3-II, p-AMPK, Atg 5, Atg 7, Atg 12, and Beclin-1 and suppressed protein levels of cyclin-dependent kinase 1 (CDK1), cyclin B, p-Akt, and p-mTOR, altogether resulting in G2/M cell cycle arrest and induction of autophagy [226,231]. Likewise, in PC12 cells, luteolin at a 100 μM concentration down-regulated expression of Beclin 1, which is an important regulator of the nucleation step, and reduced levels of LC3, which is involved in the elongation step [227]. A combination of luteolin (20 μM) and silibinin (50 μM) affected the expression of autophagy- and apoptosis-related signaling molecules in glioblastoma U87MG and T98G cells. This combination inhibited rapamycin-induced autophagy by suppressing PKCα, and induced apoptosis through the increased expression of tumor suppressor miR-7-1-3p. In addition, autophagy acted as a cell-survival mechanism in these glioblastoma cells by decreasing the expression of Beclin-1 and LC3B I and II proteins. Moreover, animal studies confirmed that luteolin and silibinin reduce tumor growth in rapamycin-pretreated mice carrying U87MG and T98G xenografts [226,227,228,229,230].

The signaling pathways regulating autophagy are still insufficiently known, but some of the signals that initiate specific responses are: (i) TOR Complex 1 (TORC1) and Ras/Protein Kinase A (PKA) pathway; (ii) Insulin-Like Growth Factor-1 (IGF-1) pathway; (iii) Damage-Regulated Autophagy Modulator (DRAM) and p53 pathway; (iv) protein kinases, and (v) Forkhead Box Class O (FoxO). These processes are discussed in detail in recent review papers [219,224,231,232].

### 3.5. Regulation of Inflammatory Pathways by Propolis and Its Polyphenolic/Flavonoid Compounds

Inflammation is a complex process caused by the overproduction of ROS, local disturbance of blood circulation, and impaired tissue metabolism, among other factors. Cancer-induced inflammation has an acute and chronic stage. Acute inflammation is the primary stage of inflammation mediated by innate immunity. It activates the immune system, lasts shortly, and is generally perceived as a therapeutic inflammation. If the inflammation continues for a long time, the second stage, chronic inflammation, begins [233,234]. Chronic inflammation is associated with diverse chronic conditions, including cancer, diabetes, obesity, and cardiovascular, pulmonary, and neurologic diseases [234]. Clinical and epidemiologic studies have indicated that chronic inflammation is involved in tumor initiation, promotion, and progression [233,234].

The interplay between inflammation and cancer implicates inflammatory mediators such as NF-κB, TNF, inducible nitric oxide synthase (iNOS), cyclooxygenases (COX), and lipoxygenases (LOX) [235,236]. Constitutive COX-1 and inducible COX-2 are key prostaglandin-producing isoenzymes. COX-2 is expressed in response to inflammatory stimuli and has an important role in cancer development and progression. Accordingly, its inhibition may suppress tumor growth, proliferation, angiogenesis, metastasis, and inflammation, and trigger apoptosis.

**Figure 2:**
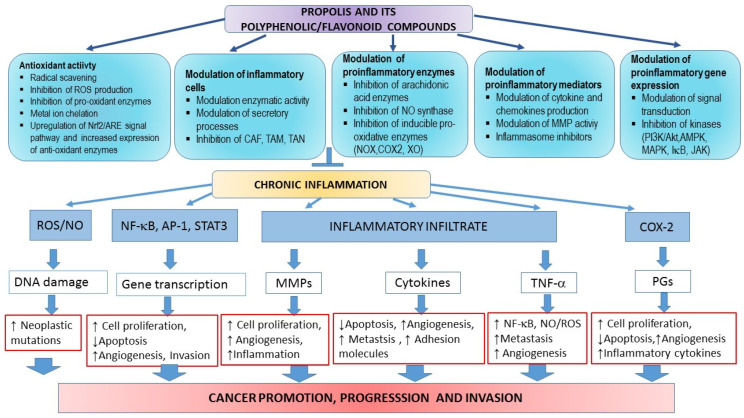
Potential therapeutic strategies to treat cancer promotion and progression by propolis and its polyphenolic/flavonoid compounds. The role and possible mechanisms of action of propolis and its flavonoids in regulation of inflammatory pathways by (i) antioxidative and radical scavenging activities, (ii) regulation of cellular activities of inflammatory cells, (iii) modulation of the enzymes involved in arachidonic acid metabolism (phospholipase A2, cyclooxygenase, and lipoxygenase) and nitric oxide synthase, (iv) modulation of the release of other pro-inflammatory molecules, and (v) transcriptional modulation of proinflammatory genes are shown. Note: ROS, Reactive oxygen species; NO, Nitric oxide; NF-κB, Nuclear factor kappa-light-chain-enhancer of activated B cells; COX-2, Cyclooxygenase-2; MMPs, Matrix metalloproteinases; TNF-α, Tumor necrosis factor-alpha; PGs, Prostaglandins; CAF, Cancer-associated fibroblast; TAM, Tumor-associated macrophages; NOX, Nicotinamide adenine dinucleotide phosphate (NADPH) oxidases; XO, Xanthine oxidase; PI3/Akt, Phosphatidylinositol 3-kinase (PI3K)/protein kinase B (AKT); AMPK, AMP-activated protein kinase; IκB, Nuclear factor of kappa light polypeptide gene enhancer in B-cells inhibitor; JAK, Janus kinases; AP-1, Activator protein 1; STAT3, Signal transducer and activator of transcription 3.

On the other hand, one of the biggest challenges in cancer treatment is the ongoing autocrine and paracrine activation of proinflammatory transcription factors, such as NF- κB, signal transducer and activator of transcription 3 (STAT-3), activator protein 1 (AP-1), forkhead box protein M1 (FOXM1), and hypoxia-inducible factor 1α (HIF-1α) that drive the overproduction of diverse mediators of inflammation—inflammatory cytokines, chemokines, cellular adhesion molecules, anti-apoptotic molecules, and iNOS [237,238,239,240,241,242,243,244,245,246,247,248,249,250,251,252]. Regarding propolis and its components, numerous studies have demonstrated the anti-inflammatory effects of various flavonoids [1,2,3,4,38,174,198,237,238,239,240,241,242,243,244,245,246,247,248,249,250,251,252]. The anti-inflammatory properties of several flavonols (quercetin, rutin, and morin) and flavanones (hesperetin and hesperidin) were examined in animal models of acute and chronic inflammation. These studies revealed several mechanisms (Figure 2) underlying the anti-inflammatory effects of propolis and its flavonoids: (i) antioxidative and radical scavenging activities, (ii) regulation of cellular activities of inflammatory cells, (iii) modulatory effects on the activities of enzymes involved in the metabolism of arachidonic acid (phospholipase A2, COX, and LOX) and NOS, (iv) adjustment of the production of other proinflammatory mediators, and (v) transcriptional regulation of proinflammatory genes [2,3,4,13,211].

The other molecular mechanisms underlying the anti-inflammatory action of flavonoids include suppression of proinflammatory enzymes, such as COX-2, LOX, and iNOS, suppression of NF-κB and AP-1, activation of phase II antioxidant detoxifying enzymes such as glutathione peroxidase (GPx), heme oxygenases, γ-glutamylcysteine synthetase (γ-GCS), superoxide dismutase (SOD) and glutathione reductase (GR), and modulation of MAPKs, PKC, and nuclear factor-erythroid 2-related factor 2 (Nrf2) [237,238,239,240,241]. Nrf2 is the transcription factor involved in both the constitutive and inducible expression of anti-oxidant responsive element (ARE)-regulated genes. Its induction by flavonoids inhibits adhesion of monocytes to endothelial cells and transmigration via suppressed expression of monocyte chemoattractant protein-1 (MCP-1) and vascular cell adhesion molecule-1 (VCAM-1), and attenuates activation of the p38 MAPK signaling pathway.

The molecular mechanisms of cytokine-modulating activities of polyphenols, including the suppression of transcription factors NF-κB and AP-1 and inhibition of MAPK activity, are particularly important mechanisms of action from the anti-inflammatory perspective. Flavonoids are effective in downregulating the expression of various proinflammatory cytokines/chemokines (TNF-α, IL-1β, IL-6, IL-8, and monocyte-chemoattractant protein-1) in different cell types such as RAW macrophages, Jurkat T-cells, and peripheral blood mononuclear cells. Furthermore, propolis and its components, CAPE, caffeic acid, quercetin, and naringenin among others, reduce the production of eicosanoids, whose increased production has been implicated in inflammation. In fact, propolis components inhibited the LOX pathway of arachidonic acid metabolism, of which CAPE was the most potent modulator [11,97,119,242,243,244]. Moreover, the inhibitory effects of CAPE on the production of proinflammatory cytokines IL-1β, TNF-α, and MCP-1 in lipopolysaccharide (LPS)-stimulated RAW264.7 macrophages have been reported [241,242].

Numerous literature data provide evidence of anti-inflammatory action of propolis [38,44,45,243,244,245]. Several studies have shown that propolis acts as a powerful anti-inflammatory agent against both acute and chronic inflammation [246,247,248]. The observed anti-inflammatory effects of propolis are attributed to the presence of flavonoids, especially galangin. Galangin inhibits COX and LOX activity, and reduces PGE2 release and the expression of the inducible isoform of COX (COX-2) [161,249,250]. Besides galangin, many authors [240,251,252,253,254] have emphasized the anti-inflammatory contribution of quercetin. For example, quercetin attenuated TNF-α-mediated induction of IL-8 and MCP-1 expression by inhibiting the activation of NF-κB. Wadsworth et al. [255] suggested that quercetin possesses the unique ability to suppress the transcription of TNF-α by inhibiting the activation of the stress-activated protein kinase (SAPK/JNK) signaling, therefore inhibiting the binding of transcription factor AP-1 to DNA. Quercetin also inhibited the phosphorylation of ERK1/2 and activity of p38 MAPK. Of note, these kinases have important roles in the post-transcriptional regulation of TNF-α mRNA.

#### Propolis and Functional Activation of Macrophages in Cancer

Macrophages are specialized cells of the immune system that secrete various inflammatory mediators. These include various growth factors, cytokines, proteolytic enzymes, proteoglycans, lipid signaling molecules, and prostaglandins [23,74,98,253,254]. Macrophages are critically involved in the initiation, maintenance, and resolution of inflammation. They are also important for the maintenance of tissue homeostasis [73,98,254]. Along with other inflammatory cells, macrophages release a broad spectrum of bioactive molecules that can induce large changes at the site of inflammation by interacting with epithelial, mesenchymal, and vascular endothelial cells. In addition, macrophages are plastic cells able to adjust their functional response to diverse stimuli by polarizing to functionally and phenotypically different states that are usually divided into two groups, classically activated (M1) and alternatively activated (M2) macrophages. M1 macrophages exert proinflammatory and anticancer functions, whereas M2 macrophages and the related tumor-associated macrophages (TAMs) regulate tissue remodeling, support angiogenesis, facilitate tumor proliferation, and may show immunomodulatory activity by releasing immunosuppressive molecules [73,253,254].

**Figure 3:**
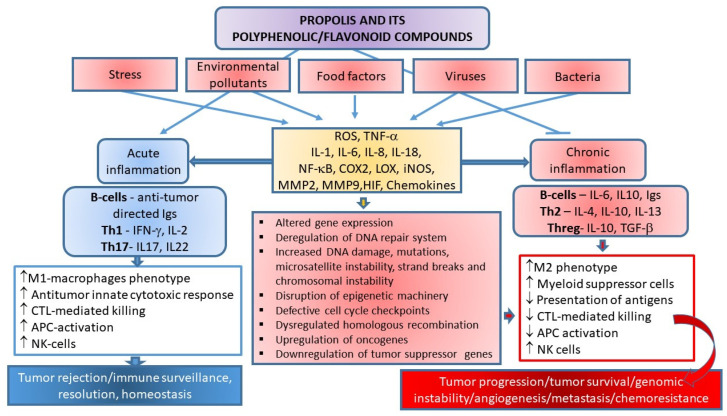
Relationship between ROS, inflammation, cancer, and propolis compounds. Increased oxidative stress can result from numerous endogenous and exogenous factors. An increase in ROS levels and a redox imbalance stimulate intracellular signaling cascades that can potentially stimulate different states of inflammation and their role in tumorigenesis. Under physiological conditions, phagocyte-derived oxidants have a protective function by disabling invading bacteria and parasites, destroying the damaged cells. Controlled, physiological inflammation is a useful, adaptive response that plays an important role in protection against infection, tissue repair, and adaptation to stress, more exactly, in the establishment of disturbed homeostasis. Chronic inflammation is linked to various steps of tumorigenesis, including cellular transformation, progression, survival, proliferation, invasion, angiogenesis, and metastasis. Propolis and its components scavenge reactive oxygen intermediates or prevent their formation, and thus reduce DNA damage, mutagenesis, and the transformation of normal cells into tumor ones. In addition, natural ingredients of propolis induce a prominent reduction in CD4 + CD25 + FoxP3+ regulatory T cells that suppress antitumor activity by IL-10 or TGF-β production. Inhibition of the M2 phenotype and stimulation of the M1 macrophage polarization associated with the production of Th1 cytokines such as TNF-α, IFN-γ, and colony-stimulating factor 2 (CSF2), increase antigen-presenting capacity, as well as the production of ROS and cytokines (IL-6, IL-12, IL-23, and TNF-α) that both contribute to the microbicidal and pro-inflammatory activities of M1 macrophages, which are termed as “fighting” macrophages. M1 macrophages are associated with a good prognosis in the cancer context. Note: ROS, Reactive oxygen species; TNF-α, Tumor necrosis factor-alpha; IL, Interleukin; NF-κB, Nuclear factor kappa-light-chain-enhancer of activated B cells; COX-2, Cyclooxygenase-2; LOX, Lipoxygenase; iNOS, Inducible nitric oxide synthase; MMP2, Matrix metalloproteinase2; MMP9, Matrix metalloproteinase 9; HIF, Hypoxia-inducible factor; Th1, T helper 1 cells; Th2, T helper 2 cells; Th17, T helper 17 cells; Threg, Regulatory T cells; TGF-β, Transforming growth factor β; Igs, Immunoglobulins; CTL, Cytotoxic T lymphocytes; APC, Antigen-presenting cell; NK, Natural killer cell.

It has been shown that ROS production is important for both the activation and functions of M1 macrophages, and the differentiation of M2 macrophages and TAMs. Antioxidant agents blocked the differentiation of TAMs and tumorigenesis in mouse models of cancer [73]. Although the role of macrophages in tumors is still a matter of debate, many data suggest that macrophages are important players in tumor development. An increased number of TAMs usually indicates a poor prognosis as TAMs promote the production of various growth factors, proteases, angiogenic factors, and ROS, which may support cancer progression (Figure 3). TAMs recruited to the tumor site have an important role in the angiogenic switch and malignant transformation, thus correlating tumor progression with tumor angiogenesis [256].

Apart from TAMs, ROS are another contributing factor with an important role in tumor biology. ROS promote carcinogenesis and malignant progression of tumor cells by various mechanisms. They induce oxidative DNA damage and genetic instability, acting as signaling intermediates that transduce mitogenic and survival messages initiated by growth factor receptors and adhesion molecules, facilitate cell motility, and remodel the tumor environment by stimulating inflammation (repair) and angiogenesis [73,98,256,257,258].

Oršolić et al. [73] demonstrated that the immunomodulatory activity of caffeic acid (an important active component of propolis), its effect on increased M1-mediated antitumor efficacy, and reduced M2 activity of TAMs, are the key mechanisms involved in the inhibition of angiogenesis and tumor growth. The inhibition of the M2 activity of TAMs decreases the activity of arginase-1 and maintains levels of NO in caffeic acid-treated mice. Previously, it has been shown that the increased activity of arginase-1 may stimulate tumor growth by attenuating the cytotoxic effect of NO. A similar effect was demonstrated by Shiri et al. [236] for curcumin. Shiri et al. [237] showed that curcumin, delivered in the form of dendrosome nanoparticles, suppresses the proliferation of metastatic breast cancer in mice by modulating the M1/M2 ratio. They found that dendrosomal curcumin increases the gene expression of STAT4 and IL-12 in tumor and spleen tissues, probably reflecting the high levels of M1 macrophages, and decreases the gene expression of STAT3, IL-10, and arginase-1, indicating low levels of M2 macrophages.

Interestingly, it has been shown that patients with psoriasis may be at higher risk of developing cancer [259], particularly for certain types of cancer, such as lung cancer, lymphoma, and non-melanoma skin cancer. Of note, Croatian propolis improved psoriatic-like skin lesions induced by irritant agents n-hexyl salicylate or di-n-propyl disulfide by decreasing the extent of lipid peroxidation and the number of inflammatory cells in the skin and peritoneal cavity, particularly by inhibiting the functional activity of macrophages [45].

In addition, TAMs can stimulate metabolic pathways participating in the inhibition of the adaptive immune response. The infiltration of inflammatory cells and an environment enriched in various cytokines (TGF-β, IL-6, IL-10, granulocyte-macrophage colony-stimulating factor (GM-CSF), IL-1β, IL-23, and TNF-α) and chemokines (“chemotactic cytokines”) may be utilized by tumor cells. These inflammatory molecules have been linked to the formation of suppressive immune cells, including myeloid-derived suppressive cells, Treg, TAMs, and their effectors, which are utilized and promoted by the tumor microenvironment [260,261]. As TAMs are the main regulators of inflammation in the tumor microenvironment, they are considered a key component of pathways connecting inflammation and cancer. This relies on their ability to produce various growth factors for epithelial and endothelial cells, and pro-inflammatory cytokines and chemokines that promote tumor survival, proliferation, and invasion. Moreover, immunosuppressive mediators released by local inflammatory or tumor cells may attenuate the host antitumor response and promote tumor progression [258,262].

## 4. Antitumor Activity of Propolis and Its Polyphenolic/Flavonoid Compounds In Vivo

Propolis demonstrated anti-tumor effects against brain, head and neck, skin, breast, liver, pancreas, kidney, bladder, prostate, colon, and blood cancers. Propolis and its flavonoid components produce pharmacological effects in chemoprevention acting by multiple mechanisms, including: estrogenic/antiestrogenic activity, antiproliferative activity, induction of cell cycle arrest or apoptosis, attenuation of oxidation, induction of detoxifying enzymes, regulation of the host immune system, anti-inflammatory and action and modulation of signal transduction [3,4,5,6,7,9,10,11,12,13,46,47,48,49,50]. Chan and co-authors [17] summarized the possible anticancer activities of propolis into the following mechanisms: (i) suppression of cancer/precancerous cells proliferation due to immunomodulatory activity; (ii) reduction in the cancer stem cell populations; (iii) inhibition of specific oncogene-related signaling; (iv) antiangiogenic effects; (v) modulation of the tumor microenvironment; and lastly, (vi) as a complementary treatment to mainstream anticancer therapies. As an advantage, the multistage nature of carcinogenesis enables intervention at each stage of the process. This may include inhibition of the formation or uptake of carcinogens, scavenging of activated carcinogens, protection of DNA nucleophilic sites, prevention of DNA-carcinogen adduct formation, modification of the activities of xenobiotic-metabolizing enzymes and other enzymes, and antioxidative activity and others [3,4,5,6,7,9,10,11,12,13,17,46,47,48,49,50]. Multiple steps of carcinogenesis may be inhibited by dietary polyphenolics/flavonoids and possible molecular mechanisms of their biological actions are shown in Table 2 and Table 3. As 90–95% of all cancers are attributed to lifestyle, and dysregulation of more than 500 different genes has been causally involved in most cancers [2], multi-targeted agents, such as propolis, are considered particularly promising in the prevention and treatment of cancer.

As already mentioned, the therapeutic activities of propolis are mainly attributed to the presence of flavonoids [3,9,10,11,12,13,14,15,16,17]. Many lines of evidence suggest that immunomodulation by propolis and its polyphenolic/flavonoid components is an important contributing factor to the antitumor and antimetastatic activities of these compounds. Flavonoids may induce the immune system [3,16,17,21,22,23,24,25,26,27,92,214,215,216,217,218,220,221,222,223], and act as powerful free radical scavengers [28,29,30,31,32,33,34,35,36,39,40,41,42,58,63]. Accordingly, a high dietary intake of flavonoids from propolis is associated with a reduced risk of cancer at different anatomical sites [3,9,10,11,12,13,14,15,16,17,18,19,20,21].

Propolis and flavonoids may restrain the development of chemically induced cancers in animal models of lung, oral, oesophageal, stomach, colon, skin, prostate, and breast cancer (see review [3,4,5,6,7,9,10,11,12,13,14,15,16,17,37,48,49,50]). Experimental animals administered with the polyphenolic immunostimulants were resistant, to various degrees, to the later inoculation of tumor cells. They showed reduced tumor take, slower tumor growth, and prolonged survival [3,9,10,11,12,13,14,15,16,17,18,19,20,21,58,59,60,61,62]. Oršolić and colleagues [58,59,60,61,62] reported that the ethanolic extract of propolis increases the survival of Ehrlich carcinoma-bearing mice and suggested that the immune-stimulatory activity of propolis contributes to macrophage activation and stimulation of their phagocytic activity. Many authors [3,4,55,73,222] have found that various propolis components exert strong anti-inflammatory and antitumor activity. Hayashi et al. [263] showed that daily oral administration of quercetin chalcone reduces the growth of the solid colon primary tumor in mice. The oral administration of proanthocyanidins, flavonoids present in grapes, also may decrease the tumor progression and the size of cutaneous carcinomas in animals [260]. Similarly, grape seed extract significantly reduced pulmonary metastasis in mice [261,264].

In a rat model, dietary curcumin enhanced the apoptotic index in azoxymethane-induced colon tumors [207,265,266]. Rao et al. [265] demonstrated the beneficial effect of a diet enriched in curcumin on azoxymethane-induced rat carcinogenesis. Curcumin decreased the incidence of colon adenocarcinomas, and reduced tumor size and expression of mucosal and tumor PGE_2_ by over 38%. Furthermore, a gavage administration of curcumin (200 or 600 mg kg^−1^) decreased diethylnitrosamine-induced hepatic hyperplasia and inflammation, and prevented an increase in the expression of oncogenic proteins p21(ras) and p53 in the rat liver [210]. The reduced expression of cell-cycling proteins PCNA, cyclin E, and cdc2 was also observed, together with the suppression of DEN-induced NF-κB activation [210,267].

Exposure to chemical carcinogens also induces the formation of aberrant crypt foci. Following azoxymethane exposure, their number was significantly reduced by resveratrol (200 µg/kg per day for 100 d) and was accompanied by the upregulation of Bax and overexpression of p21 in the crypts [211,268]. Similarly, in dimethylhydrazine-induced aberrant crypt foci, resveratrol (8 mg/kg per day) reduced tumor incidence and the degree of histological lesions [212,269]. The same was observed for rats fed a diet containing 15% grape extract [270]. Furthermore, besides inhibiting tumor growth, resveratrol (25 mg/kg for 3 weeks) increased the apoptotic index and reduced angiogenesis in MDA-MB 231 (human breast cancer) xenografts in nude mice [271,272]. Western blot analysis revealed that resveratrol decreases the expression of MMP-2, MMP-9, fibronectin, α-SMA, pPI3K, pAKT, Smad2, Smad3, pSmad2, pSmad3, vimentin, Snail1, and Slug, and upregulates the expression of E-cadherin in MDA-MB 231 cells [273]. However, tumor growth was not prevented in melanoma cell line xenografts (B16M, A375, and Duke melanoma 738) [274,275].

Kimoto et al. [276] reported that artepillin C, the active ingredient of Brazilian propolis, demonstrates cytostatic and cytotoxic effects. They reported that intratumor injection of artepillin C exhibited a cytotoxic effect by inducing apoptosis, abortive mitosis, and massive necrosis in various human and murine cancer cells in vitro and in vivo [276,277]. Furthermore, artepillin C activated the immune system. In particular, by increasing the number of macrophages and stimulating their phagocytic phenotype, artepillin C induced lymphocytosis in peripheral blood and exerted a direct antitumor effect [277,278]. According to our results, macrophage activation induced by propolis and its polyphenolic components is probably the key effector mechanism of antitumor activity of polyphenolic compounds in vivo [3,17,18,21,22,23,24,25,26,73]. Propolis and some of its components given intraperitoneally or perorally (*po*) in doses of 50 or 150 mg/kg can stimulate macrophages and reduce the number of breast cancer metastases in CBA mice [21,23]. Macrophage activation [22,24,73] and the overproduction of NO [15] are responsible for reduced tumor size and prolonged life span of animals by 14.89 to 40.76% for water-soluble derivatives of propolis (WSDP) and caffeic acid (CA), respectively, if administered before tumor cell inoculation [60].

To investigate the direct and indirect effects of WSDP, CA, and CAPE on tumor growth, they were injected subcutaneously (*sc*) at doses of 50 or 150 mg kg^−1^_,_ and shortly after tumor cells were inoculated at the site of injection. The local presence of CA or CAPE inhibited tumor growth and increased life span by 29.30% (CA) and 51.74% (CAPE) [59], while WSDP was not so effective [72]. These findings suggest that CA or CAPE inhibited mammary carcinoma growth through different mechanism(s) when compared to WSDP. The antitumor effects of CA and CAPE rely on their ability to inhibit DNA synthesis [16] and induce apoptosis of tumor cells [58,252], which indicates their possible application against cancer in preclinical and clinical trials. Furthermore, we showed that WSDP and/or its polyphenolic compounds (50 or 150 mg kg^−1^ *po* or *ip* during 7 consecutive days) reduce tumor size, as measured by the number of cells in the peritoneal cavity in Ehrlich ascites tumor (EAT)-bearing mice [23,26]. This significant reduction in tumor cells in the peritoneal cavity and the consequent increase in the survival rate of EAT-bearing mice [23,26] suggest that active biomolecules from Croatian propolis interfere with the growth of EAT cells early during treatment, either by direct elimination of tumor cells and metabolic interactions or indirectly by tumoricidal activation of macrophages and the production of various soluble factors [15,16,21,22,23,59,60,61,73,279,280,281].

### 4.1. Immunoregulation by Propolis and Its Polyphenolic/Flavonoid Compounds as Important Effector Mechanism of Antitumor Activity In Vivo

Malignant tumors usually reduce immune functions and induce abnormalities in the peripheral blood. In particular, leukemoid reaction (an increased white blood cell count) is observed in tumor patients, even during the later stages of cancer progression. The abnormal increase in leukocytes is usually due to the increased number of neutrophils. This increased number of neutrophils reduces the number of lymphocytes in the peripheral blood, while the increase in neutrophils is usually manifested with the presence of immature cells that cannot function properly [58], hindering successful treatment.

The immune system plays an essential role in maintaining the body’s homeostasis, in part by eliminating endogenous mutated cells, such as virus-infected or tumor cells. In maintaining the body’s homeostasis, macrophage activation and activity of Th1, CTL, and NK cells activated by propolis and its flavonoid components should definitely be emphasized. In other words, immunomodulation with natural products may be considered an additional option in the prevention and cure of neoplastic diseases [15,16,21,22,23,59,60,61].

#### 4.1.1. Role of Propolis and Its Flavonoids in Macrophage Activation and Polarization

An important marker of the functional capacity of macrophages is their spreading activity, which is characterized by an increased ability of adhesion and phagocytosis. Our results on macrophage spreading showed that treatments with WSDP or its polyphenolic constituents significantly increase spreading ability and consequently enhance phagocytic functions of macrophages on mutated and virus-infected cells [28]. Mononuclear cells (MN), primarily macrophages, are the main cells for tumor destruction [15,21,22,23,24,25,26,27,28,73,279,280,281]. As the most prominent antitumor effect was achieved by WSDP, as compared with the effects of its main components, it is likely that the antitumor activity of the WSDP results from the synergistic activities of polyphenolic compounds present in WSDP. Macrophage stimulation may induce the production and release of diverse cytokines such as IL-1, IL-6, IL-8, TNF-α [61,280,281], and NO [15]. Some cytokines may exert direct cytotoxic effects on tumor cells, while others affect natural killer (NK) cells and cytotoxic T lymphocytes and stimulate their activities [61]. Both our and studies of others showed that the immunomodulatory effect of propolis on murine peritoneal macrophages comes together with an increased tumoricidal action, and the stimulation of the lytic activity of NK cells. Moreover, the increased production of IL-1β by macrophages from propolis-treated mice might be related to the enhanced proliferation of T and B lymphocytes. In addition, cytokines may promote the production of antibodies, C-reactive protein, and complement factor C3 that can opsonize tumor cells [3,28,61] and activate antibody-dependent cellular toxicity. Taken together, propolis may increase the phagocytic index, NO production, and production of IgG antibodies. A combination of these effects might interfere with tumor growth and result in the elimination of tumor cells. Hence, it is likely that WSDP and its polyphenolic constituents may trigger diverse host defense mechanisms, and that destruction of tumor cells may be the net result of the previously described mechanisms.

According to many studies [22,24,25], short-term administration of propolis improves the immune system and increases the immunological response. It has been found that propolis treatment for 3 days increases the cytotoxic activity of NK cells against murine lymphoma. On the other hand, Missima and coauthors have reported that the administration of propolis for 14 days inhibits the production of IL-1β and IL-6 in C57BL/6 mice [282].

Similar to WSDP, the antitumor activity of CA results from the synergistic action of multiple mechanisms by which CA modulates proliferation, angiogenesis, immunomodulation, and viability. We demonstrated the direct effect of CA on the inhibition of cancer-induced M2-like macrophage programming, and its ability to stimulate T cell-mediated anti-tumor responses and increase the immunotherapy efficacy in EAT tumors [70]. CA may enhance the cytotoxic properties of M1 macrophages and suppress tumor growth. Inhibitory effects on TAMs may be achieved through the antioxidative activity of CA.

Based on our data, CA enhances the functional capacity of macrophages, especially Th1, and the production of proinflammatory cytokines such as IL-2, IFN-γ, and IL-12, thus reinforcing the tumoricidal activity of macrophages. IL-12 is an important proinflammatory mediator involved in the production of IFN-γ (by activating Th1 cells, cytotoxic CD8 cells, and NK cells) and the differentiation of Th1 cell populations (Figure 3). Apart from stimulation of the Th1 immune response by IL-12 and IFN-γ positive feedback regulation of IL-12 in macrophages, IL-12 promotes the production of antibodies that further activate the complement system and opsonize tumor cells. This makes tumor cells more sensitive to the cytotoxic activity of myeloid and NK cells, ultimately enhancing tumor death. CA also inhibits the formation of TAMs and M2-like macrophages, suppresses their beneficial effect on angiogenesis and tissue remodeling, and reverses their immune-suppressive and restores their anti-tumor and cytotoxic effects. The immunomodulatory activity of CA and its effect on the stimulation of M1 tumoricidal efficacy of TAMs and inhibition of M2 tumor activity of TAMs are likely the main mechanisms contributing to the inhibition of angiogenesis and tumor growth. The inhibitory effect on the M2 activity of TAMs was confirmed by the downregulation of arginase 1 in macrophages of the peritoneal cavity and stable levels of NO in the CA-treated mice. Increased arginase activity may stimulate tumor growth via different mechanisms including a reduction in NO-mediated tumor cytotoxicity [283], increased cellular proliferation due to modified polyamine and proline synthesis, deregulation of the T cell receptor (TCR) signaling, and subsequent unresponsiveness of CD8+ T cell [284], and an increased ability of myeloid suppressor cells to inhibit proliferation of T cells [285]. Thus, CA may reduce the immunosuppressive activity of arginase 1 in macrophages of the spleen and the peritoneal cavity and prevent inhibition of NO-mediated cytotoxicity in tumor cells.

Zhong et al. [286] demonstrated that polarization of the M2 phenotype is implicated in the development of colorectal cancer. Isoliquiritigenin (ISL), a flavonoid found in Brazilian propolis, may prevent azoxymethane-induced colon carcinogenesis in animals and in a mouse model of colitis-associated tumorigenesis provoked by azoxymethane/dextran sodium sulfate. Intragastric administration of ISL for 12 weeks significantly reduced the incidence of colon cancer, its multiplicity, and tumor size [287]. Moreover, ISL inhibited the M2 macrophage polarization of RAW264.7 cells and mouse peritoneal macrophages together with the inhibition of PGE2/PPARδ and IL-6/STAT3 signaling.

In yet another study [282], it was shown that propolis stimulates the production of Th1 cytokines in melanoma-bearing mice. The authors speculated that the synergistic effect of IFN-γ and proinflammatory cytokines may underlie the inhibition of tumor growth in vivo by stimulating the production of antiangiogenic factors. Of note, a robust Th1 response is necessary to destroy tumor cells, whereas a Th2 response would provide a tolerogenic environment that supports melanoma growth [276].

#### 4.1.2. Propolis, TLRs, and Immune System

Toll-like receptors (TLRs) are transmembrane proteins that recognize various conserved pathogen-associated molecular patterns (PAMPs) and have an important role in host innate immunity and the further activation of adaptive immunity. For example, the activation of TLR-2 and TLR-4 receptors is an important step against bacterial infections but also represents an important immunomodulatory response in antitumor immunity against microbial-induced tumors. Some data indicate that activated TLRs may be a target in tumor therapy [115], while others consider that activated TLRs have a negative effect and are involved in promoting tumor growth. The anticancer activity is related to the activation of TLRs on the immune cells, especially monocytes, macrophages, and dendritic cells (DCs) as a boost therapy by activating CD8+ T-cells and NK cells [288,289]. However, in addition to the activation of immune cells, TLRs are also found on the stromal cells of the tumor microenvironment such as cancer-associated fibroblasts (CAFs), TAMs, marrow-derived suppressive cells (MDSCs), and regulatory T cells (Tregs) [290,291], which can produce pro-inflammatory mediators and pro-angiogenic and growth factors, thus promoting tumor progression, metastasis, and resistance to chemotherapy and radiotherapy [291,292].

Propolis behaves as a TLR agonist and may stimulate an immune response in cancer therapy through macrophage activation and cytokine production. In particular, propolis promotes the release of TNF-α, IL-6, IL-12, MHC molecules, and IFNs, which are crucial for the activation of NK cells and cytotoxic T lymphocytes [7,293,294] and induction of tumor cell death via apoptosis, autophagy, and programmed necrosis. Numerous studies have shown that activation of TLR-4 can stimulate expression of CD40, CD80, CD86, and IL-12, which can promote the activation of T cells [295,296,297]. In addition, IL-12 also stimulates antigen presentation and promotes the cytotoxic activity of CD8+ T cells and Th1 response in immune cells [73,298].

It is likely that propolis activates the initial steps of the immune response and modulates the mechanisms of innate immunity by increasing the production of proinflammatory cytokines and expression of TLR-2 and TLR-4 by peritoneal macrophages and spleen cells. It has been shown that TLRs-mediated immunomodulatory action of propolis on human monocytes is, at least in part, due to the presence of cinnamic acid. Cinnamic acid upregulated TLR-4 expression and downregulated TLR-2, HLA-DR, and CD80 expression at noncytotoxic concentrations [7,299]. The mechanism of this response is probably based on the induction of the downstream signaling cascade that promotes the transcription of proinflammatory genes, antiviral responses, and maturation of dendritic cells, and by enhancing phagocytic activity and the ability of presenting antigens to T cells [288,300]. Anticancer and immunomodulatory activities of propolis, CAPE, and cinnamic acid derivatives have been confirmed also by TNF-α production in endothelial cells, and luminol-enhanced chemiluminescence of neutrophils. Similarly, the immunostimulatory effect of CAPE could be assigned to the increased phagocytic activity and increased ratio of CD4 to CD8 T cells [301,302,303]. CAPE also exerts robust anti-inflammatory effects by suppressing T cell activities, and through the inhibition of the store-operated Ca^2+^ entry channels and K^+^ channels, which may account for the activation of immune parameters [304].

#### 4.1.3. The Dual Role of TLRs Activation with Propolis and Its Components in Tumor Inhibition and Growth

It is known that inflammatory responses have a critical influence at various stages of tumor development [115,288,289]. As mentioned previously, TLRs are also found on the stromal cells of the tumor microenvironment [290,291]. Moreover, TLR-4 can be expressed in diverse tumors such as liver, lung, breast, gastrointestinal, and pancreatic cancer. For example, recent evidence suggests that TLR-4 is over-expressed in most clinical breast cancers and involved in breast cancer development and progression. TLR-4 may cause dysfunction of the immune response, resulting in tumorigenesis [305,306,307]. As already mentioned, the activation of TLR-4 may act as a double-edged sword in cancers; that is, TLR-4 activation has been involved in both cancer suppression and cancer growth [307]. Thus, Chang et al. [115] investigated the role of ethanol extract of Chinese propolis (EECP) and its major constituent—CAPE on inflammation-induced tumor and their effects on the TLR-4 signaling pathway, which have an essential role in breast cancer MDA-MB-231 cell line. They found that EECP and CAPE inhibit the proliferation of MDA-MB-231 cells in an inflammatory (LPS-stimulated) microenvironment by inducing apoptosis and autophagy, and through the inhibition of the TLR-4 signaling pathway. In particular, EECP and CAPE inhibited TLR-4 signaling pathway molecules such as TLR-4, MyD88, IRAK4, TRIF, and NF-κBp65, which might be one of the underlying mechanisms of the inhibition of breast cancer cells proliferation and survival. They concluded that EECP and CAPE may be promising in the treatment of inflammation-induced tumors through the induction of cell death by autophagy or apoptosis. Both Chinese propolis and CAPE activated the executioner caspase 3 in LPS-stimulated breast cancer cells, likely induced either through the autophagy or suppression of TLR-4 signaling.

Furthermore, it has been shown that CAPE interrupts the interaction of LPS with the receptor complex TLR-4/MD-2 and, therefore, inhibits the activation of TLR-4 [243]. CAPE also suppresses the production of TNF-α and IL-8 (applied at a dose of 0.1–25 μg/mL), leading to the downregulation of NF-κB, COX-2, and AP-1, and decreases the infiltration of monocytes and neutrophils. As the prolonged activation of TLR-4 during microorganism’s invasion increases oncogenic potential in the host via chronic activation of NF-κB and COX-2, it is likely that tumor progression could be inhibited by CAPE. For example, the upregulation of TLRs by LPS and other microbial products can activate the NF-κB, c-Jun/JNK, and JAK/STAT3 pathways, which all regulate cell proliferation and immunosuppression. The TLR-induced activation of key signaling pathways NF-κB and STAT3 promotes cancer pathogenesis in colon cancer, pancreatic cancer, hepatocellular carcinoma, and others [308]. In addition, NF-κB signaling may increase glycolytic energy flux during acute inflammation [309]. However, it should be pointed out that various cancers can have different degrees of TLR-4 contribution in tumorigenesis or tumor progression [288,289,290,291,292].

In addition to apoptosis and autophagy, cancer cells can die from necrosis. Although necrosis is a form of cell death, it may lead to the progression of tumor growth through the TLR-4-dependent mechanism. Namely, necrosis-induced cell death is related to the release of danger-associated molecular patterns (DAMPs). Certain DAMPs, such as a high mobility group box-1 protein (HMGB1), can potentially promote cancer progression. Cancer cells with a high expression of HMGB1 may induce cancer cell invasion, migration, and metastasis, and a proliferation of endothelial cells in vitro and neovascularization in vivo. It has been revealed that DAMP-derived molecules in the tumor microenvironment induce chronic inflammation via TLR-4 [306,307]. Another study found that the TLR-4/MD-2 complex promoted the development of hyperpermeable regions by increasing expression of C-C chemokine receptor type 2 (CCR2) in inflamed mice, and consequently the rate of lung metastasis. Metastases were initiated through the TLR-4-dependent NF-κB inflammatory pathway [306,307]. All these findings suggest the critical role of the TLR-4/MD-2 complex in inflammation-associated cancers, which, as mentioned before, can be interrupted by CAPE.

#### 4.1.4. Role of Propolis and Its Polyphenolic/Flavonoid Compounds on Chronic Viral or Bacterial Infections

Many malignancies develop at the site of chronic infection. A cause of infection may range from environmental factors to viruses or bacteria. Some microorganisms associated with cancer development have mechanisms that prevent normal pathways involved in maintaining the integrity of genetic information, preventing apoptosis of damaged cells, and consequently reducing the ability to repair damage, causing cell transformation, unwanted cell proliferation, and reduced response to therapy. Different viruses can affect different stages of tumor formation, regardless of their specificity. It is estimated that infection contributes to 20% of all cancer cases diagnosed in the world [310]. For example, human papilloma viruses, hepatitis B virus (HBV), hepatitis C virus (HCV), Epstein–Barr virus (EBV), and human T-cell leukemia virus type1 (HTLV-1) are recognized as risk factors for the development of cervical cancer, hepatocellular carcinoma, lymphoproliferative disorders, and adult T-cell leukemia/lymphoma (ATL), respectively. The pathological effects of these viruses are related to inflammatory mechanisms and inhibition of tumor suppressor proteins [310]. It is known that innate immunity (monocytes/macrophages, neutrophils, dendritic cells (DCs), and NK cells) is the first line of defense against infectious agents, with a tremendous role in their early recognition and formation of a pro-inflammatory environment. The process of starting the immune response is based on the recognition of the microbes’ conserved structures or damaged cell products, known as PAMPs and DAMPs, respectively, by cellular proteins called pattern-recognition receptors (PRRs). PRRs with potential antiviral responses are TLRs, C-type lectin receptors (CLRs), retinoic acid-inducible gene (RIG)-I-like receptors (RLRs), NOD-like receptors (NLRs), and DNA sensors. Activation of circulating monocytes and macrophages, which have a prominent role in protection against viruses, may occur via TLR-dependent and -independent pathways. TLRs-mediated mechanisms promote the production of proinflammatory cytokines, adhesion molecules, chemokines, immunoreceptors, and interferons (IFNs) that may be helpful in antiviral immunity.

The anti-microbial, anti-bacterial, and anti-viral effects of propolis and its components are well known. Propolis and its flavonoids, such as quercetin, galangin, chrysin, and derivatives of hydroxycinnamic acids (e.g., caffeic acid, cinnamic acid, and p-coumaric acid), stimulate macrophages to induce activation of Th1 cells, which promote the cellular immune response. Th1 cells secrete cytokines such as IFN-γ, TNF, and IL-2, which are key coordinators of the immune response to intracellular pathogens. IFN-γ activates macrophages and DCs, enhancing their ability to neutralize intracellular pathogens and to present antigens to T lymphocytes, whereby the secretion of TNF, lymphotoxin, and IL-2, contributes to antimicrobial defense.

The mechanism by which propolis and related flavonoids act on microorganisms depends on their chemical composition and includes diverse mechanisms of action. In some cases, their effects rely on the hydrophobicity or lipophilicity of the flavonoids that penetrate the lipid bilayer of the cell membrane and make the cells more permeable, resulting in leakage of the intracellular content. Some compounds may cross the microbial cellular membrane, but interactions with membrane enzymes and proteins may induce an opposite leakage of protons, impairing various aspects of cellular activity, such as membrane-coupled energy production, membrane transport, and other metabolic regulatory functions, DNA and RNA synthesis, and protein translation [311]. Furthermore, they may deteriorate cell wall and cytoplasmic membranes, induce leakage of cellular components, and modify fatty acid and phospholipid constituents.

The antiviral activity of flavonoids is based on numerous targets that are also known targets of some antiviral drugs. Flavonoids interfere with some proteins and enzymes involved in viral replication. The main antiviral mechanisms of propolis and its flavonoids are based on: (i) inhibition of virus transcription; (ii) inhibition of viral polymerases including RNA polymerase, DNA polymerase, and reverse transcriptase; (iii) binding of flavonoids to nucleic acids or capsid proteins; (iv) inhibition of virus–cell interactions; and (v) and stimulation of the immune system (INF-α,β,γ) [298,312].

Numerous studies have shown that the viral proteins of oncogenic viruses exhibit their carcinogenic effect by modulating signaling pathways. HTLV-1, an RNA tumor virus and one of the most potent oncogenic agents, causes about 5% of ATL leukemia due to changes in regulatory proteins, including Tax and Rex. These regulatory proteins are sensitive to propolis, quercetin pinocembrin, myricetin, and CAPE, probably because these compounds prevent the action of TAX oncogene in the activation of the NF-κB pathway [310,312].

Furthermore, an RNA virus from the HIV family suppresses the immune system and decreases the ability of the body to fight against viral infections, progressing to the three most common forms of cancer known as “acquired immunodeficiency syndrome (AIDs)-defining cancers” or “AIDS-defining malignancies”, which include Kaposi sarcoma, aggressive B-cell non-Hodgkin lymphoma, and cervical cancer. Flavonoids (chrysin, acacetin, and apigenin) successfully inhibit virus entry via the receptor CD4 and co-receptors CXCR4 and CCR5, affect the viral cycle, inhibit reverse transcriptases, telomerases, glucose metabolism involved in the pathogenesis of HIV infection of T cells and macrophages, decrease inflammation and ROS level, stimulate glutathione synthesis whose deficiency is provoked by cysteine catabolism, and inhibit viral expression in a concentration-dependent environment. Furthermore, flavonoids can reverse the expression of some cellular proteins involved in certain signal transduction pathways that are modified by viral infection [310,311,312,313].

Human papillomavirus (HPV)-induced cervical cancer represents about 3% of overall cancers in women, whereas around 2% of overall cancers in men are HPV-induced. The oncogenic, “high-risk” HPVs are HPV 16, 18, 31, 33, 35. 39, 45, 51, 52, 56, 56, and 59. Development of cervical cancer is dependent on two major oncogenes *E6* and *E7*. Propolis and flavonoids have the ability to inhibit E6 and E7 proteins and increase p53 and Rb activity, induce apoptosis and cell cycle arrest, and inhibit tumor growth. Flavonoids from propolis also prevent the translocation of NF-KB and AP1 and transcription of specific genes, and prevent suppression of human telomerase reverse transcriptase (hTERT) [314].

Epstein–Barr Virus (EBV) is a DNA virus first noticed in Burkitt’s lymphoma cells. The components of Brazilian propolis can inhibit the expression of key viral transcription factors during the lytic cycle and interfere with the transactivation function of the Rta viral protein, interfering with the production of functional EBV virions [315]. Different flavonoids are thought to cooperate together, exerting a synergistic antiviral effect. In addition, flavonoids can enhance the efficacy of antiviral drugs. Therefore, flavonoids are capable to protect host cells from damage induced by viral infection.

Likewise, chronic infection with the bacterium *Helicobacter pylori* is one of the main contributors to developing duodenal and gastric ulcer diseases, gastric cancer, and gastric mucosa-associated lymphoid tissue (MALT) lymphoma, the most common cancer worldwide [315,316,317]. *H. pylori* colonizes the gastric mucosa of more than 50% of the world’s population. It is classified as a group 1 carcinogen by the International Agency for Research on Cancer [305,316]. *H. pylori* can have direct effects on gastric epithelial cells causing mutations in genes that regulate the cell cycle, and inducing defects in DNA repair mechanisms, the loss of cell adhesive properties, and epigenetic modifications that can alter cell behavior leading to cell autonomy and malignant transformation [317]. The indirect effect of *H. pylori* infection results in a chronic inflammatory response and an increased number of inflammatory cells such as neutrophils and macrophages, which overproduce reactive oxygen and reactive nitrogen species (ROS/RNS). This may result in an accumulation of mitotic errors and increased oxidative stress and DNA damage. Various dietary polyphenols demonstrate protective and therapeutic potential in peptic ulcers by: (i) stimulating expression of growth factors and prostaglandins; (ii) promoting re-epithelialization, neovascularization, and angiogenesis; (iii) reducing oxidative damage of mucosa; (iv) increasing antioxidant, antacid, and antisecretory activity; (v) suppressing release of anti-angiogenic factors; (vi) enhancing levels of endothelial NOS-derived NO; (vii) increasing endogenous mechanisms of mucosal defense; and (viii) inhibiting *H. pylori* colonization, gastric morphological changes, inflammation, and ulceration [318]. Decreased production of proinflammatory cytokines and adhesion agents, suppression of leukocyte–endothelium interactions, inhibition of nuclear inflammatory pathways, and modulation of intracellular signaling further contributes to the anti-ulcer action of dietary polyphenols [317].

As presented in Table 2 and Table 3 and Figure 2, polyphenols target multiple inflammatory components and show strong antimicrobial activities. Propolis and its flavonoid components offer protection against infection by a plethora of antimicrobial actions including bacteriostatic and bactericidal effects, reduced colonization, suppression of toxin production/reception, and downregulation of intracellular signaling components (e.g., ion channels, ADP-ribosylation), among others [27,38,39,304,305].

Propolis and its components cinnamic and caffeic acid interfere with the early events in immune response, increasing the expression of TLR-2 and TLR-4, MHC molecules and costimulatory molecules (such as HLA-DR and CD80, respectively), cytokine release, and the microbicidal activity of human monocytes [27]. According to Conti et al. [27], propolis modulates the maturation and function of dendritic cells (DCs), offering new possibilities for pharmacotherapeutic approaches in DC-based strategies and discovery of new immunomodulators.

#### 4.1.5. Associations between Flavonoid Intake, Gut Microbiota, and Cancer

Numerous literature data suggest that non-nutritious dietary ingredients, such as flavonoids, may affect the composition of the intestinal microbiota [319,320,321]. These data emphasize the relationship between flavonoid intake and diverse inflammation-associated chronic conditions such as certain forms of cancers, including colorectal, breast, lung, and liver cancer. The effect of dietary intake on gut microbiota is, at least partially, determined by the metabolism of flavonoids by the gut microbiota. In particular, gut microbiota may regulate cancer processes by affecting genetic instability, sensitivity to host immune response, progression, and therapy efficacy [322]. It has been demonstrated that gut microbiota may affect tumor development and modify interactions with the immune system. Gut dysbiosis represents an imbalance between the number and diversity of the commensal and pathogenic bacterial communities and the production of diverse microbial antigens and metabolites. As the immune system and the gut microbiota work together to preserve intestinal homeostasis, alteration of microbiota composition may lead to immune dysregulation, promoting chronic inflammation and tumor development. In addition, gut microorganisms and their toxic metabolites may reach other body parts via the circulatory system and disturb the physiological conditions of the host and the release of various bioactive molecules, which may impact inflammation and tumorigenesis in specific organs. On the other hand, specific ingredients and substances from *Lactobacillus* and *Bifidobacterium* strains demonstrated anticancer effects through their antiproliferative, proapoptotic, and antioxidant activities. Regarding antioxidative action, these strains can express and secrete GSH and antioxidant enzymes (SOD, CAT, and GPx), scavenge free radicals, release antioxidants of small molecular weight and exopolysaccharides, and chelate metal ions, preventing deleterious effects of various carcinogens. These activities reduce oxidative stress, lipid peroxidation, and oxidative damage of DNA and proteins, promote DNA repair, and may help to reduce the risk of cancer development [323].

The aforementioned dysbiosis of gut microbiota could play an important role in tumor proliferation, invasion, death by apoptosis and autophagy, and metastasis. While metabolites from local microbiota may serve as signaling molecules and modify cell-intrinsic pathways, distal gut microbiota affect tumor development by regulating the systemic immune response. For example, local dysregulation of microbiota in the lungs can stimulate the Myd88-dependent production of IL-1β and IL-23 from myeloid cells, and induce activation and proliferation of lung resident Vγ6 + Vδ1 + γδ T cells. These γδ T cells then produce IL-17, promoting infiltration of neutrophils and inflammation in the tumor microenvironment. Other upregulated factors such as IL-22, amphiregulin, and other effector molecules further may contribute to tumor cell proliferation [324]. In addition, gut microbiome dysbiosis may promote local inflammation via increased transcription of chemokine C-C chemokine ligand 5 (CCL5), which recruits many lymphocytes in the intestine. The developed inflammatory state promotes the proliferation of epithelial cells by locally activating the IL-6 pathway. In addition, the upregulation of TLRs by LPS and other microbial products can activate the NF-κB, c-Jun/JNK, and JAK/STAT3 signaling, thus contributing to cell proliferation and immunosuppression.

Several lines of evidence suggest that environmental factors, such as diet, in addition to genetic and immune determinants, may affect metabolism, immunity, and the host response to pathogens [325,326]. According to Helmink et al. [324], the gut microbiota may promote carcinogenesis by affecting steroid (estrogen) metabolism, and the ability to alter the profile of circulating estrogens and phytoestrogens. Intestinal microbes possess enzymes that deconjugate conjugated estrogen metabolites prepared for excretion and return them back into circulation. Furthermore, they are capable of degrading otherwise indigestible dietary polyphenols and synthesizing estrogen-like compounds with varied estrogenic potency. Interestingly, phytoestrogens have a much higher affinity for ERβ than ERα, which might be an evolutionary adaptation as estrogen-dependent breast cancers are usually mediated by ERα, whereas physiological effects are preferentially mediated by ERβ. It should be pointed out that some triple-negative breast cancers exhibit active ERβ. However, most epidemiological studies demonstrated a protective effect of phytoestrogens from propolis in breast cancer [261], particularly in premenopausal women; although, the results are not consistent. Current data indicate that intake of propolis induces estrogenic activity and suggest that propolis is a valuable source of phytoestrogens [122,123,130,149,150,151,327]. According to Ge et al. [309], gut microbiota can be used as a tumor marker and may ensure valuable insights into the pathophysiology of malignant tumors.

**Figure 4:**
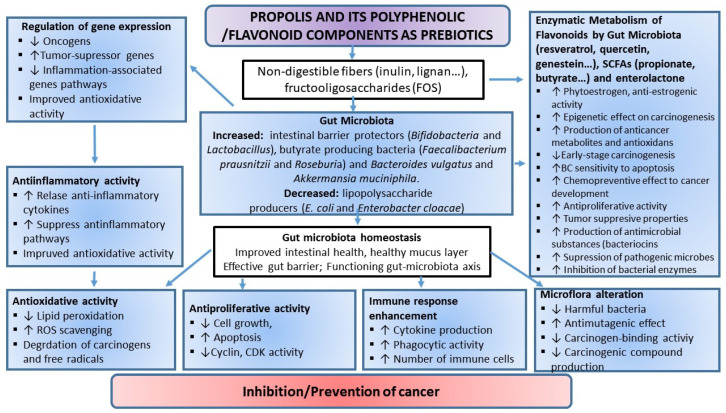
Interaction between propolis and its polyphenolic/flavonoid components and the intestinal microbiota. Propolis and its components exert a positive effect on the intestinal microbiota, leading to the establishment of intestinal homeostasis through improved gut health, healthy mucus layer, effective intestinal barrier, and functional gut–microbiota axis. The bioavailability and impact of polyphenols on the host greatly depend on their transformation by specific components of the gut microbiota. Acting as prebiotics, propolis and its polyphenolic/flavonoid components stimulate the immune system, reducing inflammatory responses and oxidative stress.

It has been shown that propolis and its polyphenolic/flavonoids have prebiotic effects, i.e., an ability to selectively stimulate the growth and metabolism of healthy bacteria, thereby improving intestinal health [325,326,327]. Propolis is rich in prebiotics that can improve the intestinal barrier and prevent the dislocation of pathogens, toxins, and specific bacterial metabolites across the gut barrier into blood [328]. Several mechanisms are speculated to be involved in cancer prevention and therapy using prebiotics: (i) modification of gut microbiota, (ii) enhancement of gut barrier functions, (iii) protective effect on DNA damage of intestinal epithelium and degradation of potential carcinogens, and (iv) stimulation of immune system and anti-inflammatory properties. Acting as prebiotics, propolis and its polyphenolic/flavonoid components stimulate the immune system, reducing inflammatory responses and oxidative stress (Figure 4).

Onur et al. [328] investigated the potential of supportive therapy with acidophilus milk (AS) and propolis extract (PE) in a mouse xenograft breast cancer model. They found that treatments with PE, AS, and their combination, reduce tumor sizes, whereas the AS and PE combination significantly enhanced the production of IFN- γ. The authors reported that the most pronounced anti-tumor effect was obtained with the combination of AS and propolis.

According to [39] and Hao et al. [171], polyphenols, such as quercetin, resveratrol, chrysin, and citrus flavonoids, have the ability to modify gut microflora and improve an imbalance of intestinal microbiota, thereby promoting gut health. Intestinal polyphenol metabolites are capable of modifying the activity of enzymes important for the detoxification process and inflammation [3,13,49,75,129,170]. Some polyphenols from the propolis also may influence bacterial metabolizing enzymes and overall cancer risk. *Lactobacillus* and *Bifidobacterium* strains may exert anti-cancer activity via several mechanisms: modification of the intestinal microbiota and its metabolism suppressed production of bacterial fecal enzymes (e.g., β-glucosidase, β-glucuronidase, and nitroreductase) that transform procarcinogens to carcinogens, binding of carcinogens and attenuated cyto- and genotoxicity of cacal/fecal content. On the other hand, supplementation with resveratrol significantly reduces the activities of fecal and host colonic enzymes β-glucoronidase, β-glucosidase, β-galactosidase, mucinase, and nitroreductase. We have shown that quercetin can increase some strains of *Lactobacilli* and *Bifidobacteria* and protect DNA from mycotoxin-induced damage.

Mechanisms underlying the anti-proliferative effects of *Lactobacilli* and *Bifidobacteria* in cancer are numerous. Following colonization of the intestinal epithelium, the bacteria and metabolites that they release may repress tumor progression through cell-cycle arrest, inhibition of proliferation, downregulation of overexpressed cyclins D1 and E1, inhibition of angiogenesis and tumor survival, and finally a decrease in polyamine synthesis that otherwise supports the growth of cancer cells [261,329,330]. These modes of action may be achieved by short-chain fatty acids, conjugated linoleic acids, and exopolysaccharides that are produced by *Lactobacillus* and *Bifidobacterium* strains.

It has been shown that interactions between the microbiota and the polyphenols are mutual. The positive effect of polyphenols on increasing the number of beneficial bacteria may in turn act on polyphenols and enhance their bioavailability. Furthermore, functional phenolic constituents released after microbial fermentation in the colon may improve colon health through their antioxidative, anti-inflammatory, and immunomodulatory activities. Similarly, reciprocal interactions between polyphenols from propolis extract and intestinal microbiota may affect metabolic pathways, resulting in health benefits and potential prevention of cancer disease development. Through these prebiotic effects, polyphenol-rich products, such as propolis, may increase the production of intestinal mucus in the host, stimulate the release of gut peptides with antimicrobial activity, and modify hepatic bile acids and gut immunoglobulin secretion, leading to protection against colon cancer and other types of cancer [180,261,265,266,267,268,269,270,328,329,331]. Probiotic strains are efficient scavengers of hydroxyl radicals and superoxide anions and are capable of producing a wide range of antioxidants, including glutathione transferase, catalase, SOD, GSH, folate, uric acid, and vitamins C, E, and β-carotene, which are absorbed and distributed through the body [332,333,334,335,336]. Antioxidative molecules from *Lactobacillus* and *Bifidobacterium* can protect the intestine, liver, and kidney against oxidative stress-associated diseases, such as cancer [332,333,334,335,336]. *Lactococcus lactis* is likely involved in increased catalase activity in the liver following the intake of polyphenolic compounds. Exopolysaccharides are released by probiotic bacteria and potentially contribute to oxidative stress attenuation by stimulating *Lactobacillus*-mediated expression of Nrf2 expression in the liver [333,336]. Therefore, the protective effect of propolis and its polyphenols against oxidative stress is partly based on its prebiotic effects and recovery of intestinal microbiota [265]. Furthermore, probiotics can inhibit intestinal pathogens and deplete postprandial lipids involved in oxidative damage. According to our results and other authors [337,338,339,340], intake of polyphenols may be effective in reducing *Enterobacteriaceae*. High levels of fecal *Enterobacteriaceae* are associated with inflammatory bowel disease, immune imbalance, and the inflammatory status of the gut epithelium [340]. In conclusion, within the gastrointestinal tract, dietary polyphenols might exert direct and indirect antioxidative effects by modulating gut microbiota. The most important mechanisms of action include the binding of prooxidant iron, ROS scavenging, and probably inhibition of COX-2 and LOX.

### 4.2. Antiangiogenic Effect of Propolis and Its Polyphenolic/Flavonoid Compounds

Angiogenesis, the formation of new blood vessels, is important for tumor maintenance and metastasis. It has a crucial role in tumor growth as it supplies oxygen and nutrients that sustain the rapid and uncontrolled proliferation of cancer cells. The process of angiogenesis is usually required for tumors whose size is beyond 1–2 mm^3^ and is predominantly stimulated by the vascular endothelial growth factor (VEGF) [341,342,343]. It is known that ROS regulates angiogenesis and tumor growth through VEGF. Both tumor and stromal cells may release proangiogenic factors, such as VEGF, which promote the formation and maintenance of new vessels [70,341,342]. It is well-known that the cancer microenvironment, consisting of stromal, endothelial, immune, and cancer cells, is very important for carcinogenesis. Natural products, including propolis and their constituents, may interfere with this symbiosis.

Oršolić et al. [70] investigated the effect of caffeic acid on tumor growth, angiogenesis, functional ability and polarization of macrophages, and oxidative stress levels. A probable functional link has been found between the antioxidative effect of caffeic acid and the different mechanisms by which caffeic acid inhibits angiogenesis and tumor growth. This includes: (i) inhibition of ROS production critical for tumor growth and angiogenesis; (ii) inhibition of VEGF production and tumor progression (iii) inhibition of the polarization of M1 phenotype into M2 phenotypes of spleen and peritoneal macrophages in mice bearing EAT, and (iv) immunomodulatory activity as evidenced by the increased M1 tumoricidal efficacy of TAMs and suppressed M2 tumor activity of TAMs. Of note, TAMs are considered as attractive candidates for novel therapeutic strategies and one of the potential approaches directed to TAMs is a reversal of their immuno-suppression activity and restoration of tumor cytotoxicity [341]. It was observed that caffeic acid downregulates VEGF levels in ascites fluid, tumor cells, and macrophage cells, and reduces the microvessel density. Animals treated with CA reduced secretion of VEGF in ascites fluid by 34.82% and 52.57% at a dose of 40 mg kg^−1^ and 80 mg kg^−1^, respectively [70]. CA treatment decreased VEGF levels in tumor cells isolated from total cells of ascites in the peritoneal cavity to 45.22% and 44.53% of control cells, while in the macrophages of the peritoneal cavity, secretion of VEGF could not be detected. Consequently, the reduction in VEGF results in reduced microvessel density. Thus, caffeic acid decreased the number of vessels by 66.14% and 73.73% at a dose of 40 mg kg^−1^ and 80 mg kg^−1^, respectively, as compared with the peritoneum of control animals. Our findings also suggested that CA may activate macrophages to secrete factors that regulate the function of T cells and other cells of the immune system [21,22,23,24,25,26,27,28]. As explained previously, macrophages are one of the major cells of innate immunity with pivotal roles in the primary response to tumor cells, preservation of tissue homeostasis, inflammation, and immunity [70,341,342,343]. Besides their important role in killing tumor cells, they can be involved in tumor growth. In particular, M1 macrophages possess anticancer activity, whereas M2 macrophages and related TAMs promote tissue repair and remodeling, angiogenesis, and tumor growth [341,342,343].

Antiangiogenic effects of propolis have also been demonstrated. CAPE, one of the major bioactive compounds from propolis, effectively suppressed the adhesion and invasion capability of human hepatocellular carcinoma cells (SK-Hep1) by inhibiting the expression of matrix metalloproteinases (MMPs) MMP-2 and MMP-9, and NF-κB DNA-binding activity [343]. Song et al. [344] demonstrated the anti-angiogenic effect of CAPE in a chick embryo chorioallantoic membrane (CAM) assay system. CAPE also suppressed VEGF secretion by MDA-MB-231 cells and the development of capillary-like tubes by endothelial cells, further indicating its anti-angiogenic potential [345,346,347,348]. Similarly, Izuta et al. [347] reported that CAPE inhibits VEGF expression in human umbilical vein endothelial cells (HUVECs). Not only CAPE, but also Brazilian propolis ethanolic extract, reduced the number of new vessels and suppressed the proliferation of HUVECs [105,349]; the antiangiogenic effect was predominantly mediated through the inactivation of the survival pathway ERK1/2 and by inducing apoptosis in tube-forming endothelial cells. Likewise, extracts of propolis containing artepillin C and CAPE decreased the formation of new vessels and expression of MMPs and VEGF in various cancer cells [350]. It was also found that some compounds from Brazilian green propolis inhibit the expression of the hypoxia-inducible factor-1 (HIF-1) protein and HIF-1 downstream targets such as glucose transporter 1, hexokinase 2, and VEGF-A [351]. HIF-1 has been appreciated as a relevant cancer drug target as there is a strong positive correlation between the level of HIF-1 and tumor angiogenic and metastatic potentials [352]. In another study, Valente et al. [352] found that a methanolic extract of Portuguese propolis was selectively toxic against malignant cells. It strongly inhibited the growth of human renal cell carcinoma (RCC) in vitro. On the other hand, the ethanolic extract of Portugal propolis (P10.EE) attenuated proliferation and migration of tumor breast (MDA-MB-231) and prostate (DU145) cells and reduced their viability, but was less cytotoxic against non-tumor cells and fibroblasts. In addition, P10.EE reduced total biomass and proliferation of human brain-microvascular endothelial cells and reduced vessel sprouting in the CAM assay. The effects of Chinese red propolis and its related products on VEGF-induced proliferation and migration of HUVECs were also investigated [345,346,347,348]. Chinese red propolis inhibited both VEGF-induced HUVEC proliferation and migration. Thus, it seems that propolis modulates the tumor microenvironment including the stromal, endothelial, immune, and cancer cells. While it directly affects cancer cells by inducing apoptosis, it is also able to prevent tumor progression through the anti-angiogenic and immunomodulatory effects.

As previously mentioned, resveratrol has been identified as a bioactive compound in Romanian propolis [99,353]. Rauf et al. [354] reviewed the effects of resveratrol, demonstrating that resveratrol may affect cell growth, inflammation, apoptosis, angiogenesis, and invasion and metastasis in vitro. Multiple molecular targets of its action have been identified: tumor suppressors p53 and Rb, cell cycle regulators (cyclins, cyclin-dependent kinases (CDKs), p21WAF1, p27KIP, and INK and the checkpoint kinases ATM/ATR), transcription factors NF-κB, AP-1, c-Jun, and c-Fos, angiogenic and metastatic factors such as VEGF and MMP-2/9, COXs, and various apoptotic and survival regulators, including Bax, Bak, PUMA, Noxa, TRAIL, APAF, survivin, Akt, Bcl2, and Bcl-XL. Moreover, resveratrol is capable of exerting pro-oxidant effects under specific experimental conditions, which may induce oxidative DNA damage, leading to cell cycle arrest or apoptosis.

Resveratrol also inhibited tumor-induced neovascularization by suppressing VEGF secretion in various cancer cell lines and in vivo models [353,355,356,357,358]. The anti-angiogenic effect of resveratrol was confirmed through the inhibition of other mediators, including the basic fibroblast growth factor (bFGF) and MMP-2/9, the endopeptidases that degrade the extracellular matrix and contribute to cell migration in angiogenesis and metastasis [356]. Resveratrol remarkably inhibited the activity and expression of MMP-9, and attenuated PI3K activity and phosphorylation of AKT and mTOR in breast cancer cells [54,355]. It also inhibited NF-κB transcriptional activity and DNA binding of NF-κB on the MMP-9 promoter, thus suggesting that resveratrol suppresses the invasion of breast cancer cells by attenuating MMP-9 and downstream PI3K/AKT and NF-κB pathways [213,214,215,216]. In addition, Uvez et al. [358] showed the inhibitory effects of resveratrol on bFGF-induced angiogenesis in the chick CAM model.

Pradhan et al. [359] coined the concept of angioprevention, pointing out the capacity of dietary phytochemicals, including resveratrol, to inhibit inflammation and angiogenesis. Together with the resveratrol-mediated suppression of NF-κB, resveratrol reduces the expression of inflammatory cytokines, such as IL-1, -6, -8, TNF-α, and VEGF as well [359]. In addition, numerous studies have shown that resveratrol suppresses the activation of MAPK signaling pathways. As MAPK signaling mediates inflammatory responses and the proliferation of endothelial cells, the inhibitory effect of resveratrol on MAPK further indicates its potential in angioprevention and tumor metastasizing.

### 4.3. Antimetastatic Effect of Propolis and Its Polyphenolic/Flavonoid Compounds

**Figure 5:**
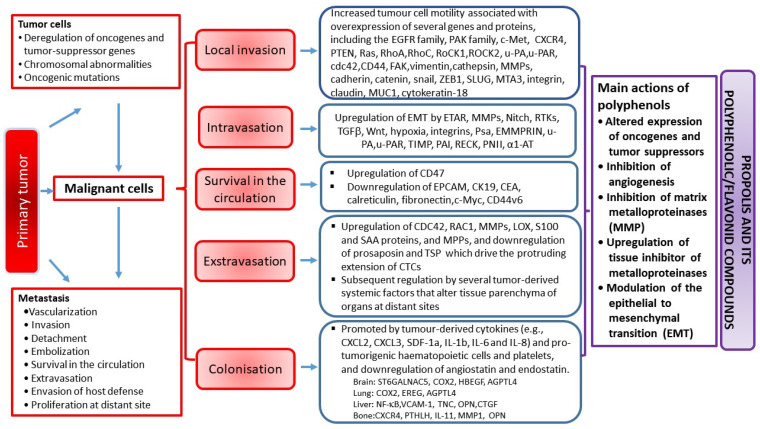
Antimetastatic effect of propolis and its polyphenolic/flavonoid compounds. Propolis and its components participate in all steps of the metastatic spread from the primary tumor through numerous mechanisms. Development of tumor cells includes survival, proliferation, invasion, angiogenesis, and metastasis. Note: EGFR, epidermal growth factor receptor; PAK, p-21 activated kinases; c-Met, tyrosine-protein kinase Met or hepatocyte growth factor receptor (HGFR); CXCR4, C-X-C Motif Chemokine Receptor 4; PTEN, Phosphatase and tensin homolog; Ras, oncogen; Rho, Rac, and Cdc42, RhoA, RhoC, Rho GTPase; RoCK, Rho-associated protein kinases; uPA, Urokinase plasminogen activator; uPAR, Urokinase plasminogen activator receptor; ECM, extracellular matrix; EMT, epithelial–mesenchymal transition; CD44, transmembrane glycoprotein also referred to as P-glycoprotein 1; FAK, Focal adhesion kinase; MMPs, Matrix metalloproteinases; EPCAM, Epithelial cell adhesion molecule; ZEB1, Zinc finger E-box-binding homeobox 1; SLUG, Zinc finger protein SNAI2; RECK, Reversion-inducing cysteine-rich protein with kazal motifs; CCL2, C-C motif chemokine ligand 2; MUC1, Mucin 1; ETAR, Endothelin type A receptor; TGFβ, Transforming growth factor β; Twist, basic helix-loop-helix factors; SDF1, stromal cell-derived factor 1; Snail, Slug, transcription factors of a snail family; TIMPs, Tissue inhibitors of metalloproteinases; PAI, Plasminogen activator inhibitor 1; MTA3, metastasis-associated protein; EMMPRIN, extracellular matrix metalloproteinase inducer; PN-II: protease nexin-II; alpha1-AT, alpha 1-antitrypsin; EPCAM, epithelial cell adhesion molecule; CK19, Cytokeratin 19; CEA, carcinoembryonic antigen; SAA, serum amyloid A; LOX, protein-lysine 6-oxidase; RAC1, Ras-related C3 botulinum toxin substrate1; cdc42, cell division control protein 42 homolog; S-100, S-100 protein; ST6GALNAC5, α2,6-sialyltransferase; AGPTL4, Angiopoietin-like (ANGPTL) protein; OPN, Osteopontin; CXCR4, chemokine receptor 4; EREG, epiregulin; COX-2, Cyclooxygenase-2; TSP, Thrombospondin; CTCs, circulating tumor cells; VCAM-1, Vascular cell adhesion molecule 1; TNC, tenascin C; CTGF, Connective tissue growth factor, NF-κB, Nuclear factor kappa-light-chain-enhancer of activated B cells; PTHLH, parathyroid hormone-like hormone.

Oršolić et al. [16] investigated the anti-metastatic effects of WSDP (Croatian or Brazilian) and related polyphenolic compounds (CA, CAPE, and quercetin). If they were given to experimental animals (50 or 150 mg/kg) prior to the inoculation of tumor cells, a significant decrease in the number of tumor lung metastases was found. Likewise, the antimetastatic effect of similar capacity was observed if WSDP and other compounds were administered after tumor cell inoculation. These compounds decreased the number of metastases, reduced the size of subcutaneous tumors, and enhanced the survival of treated animals [23,28,58]. The effect of WSDP is likely achieved by the synergistic activities of the polyphenolic components present in propolis [59]. Flavonoids such as apigenin, quercetin, luteolin, kaempferol, and genestein exhibit powerful anti-cancer properties against various in vitro and in vivo models of cancer, by regulating the critical transduction pathways involved in the migration and invasion of cancer cells and metastatic progression. These flavonoids play an important function in the regulation of metastases through the modulation of epithelial–mesenchymal transition or regulatory molecules such as MMPs, uPA/uPAR, TGF-β, TIMP-1, and TIMP-2 expression and other contributors of the complex process of metastatic spread [360,361,362,363,364,365]. Of note, although some studies have confirmed the antitumor effect of flavonoids in animal tumor models [362,366], neither of them reduced tumor growth when given prior to tumor cell inoculation. This highly indicates the potential beneficial effects of honeybee propolis as supportive therapy in patients with metastatic tumors (Figure 5). It was also found that geographical origin did not affect the biological properties of propolis [9,17].

In another in vivo murine study that evaluated metastases formation in the lung after *iv* injection of mammary carcinoma cells [31], water-soluble Croatian or Brazilian propolis extracts were administered either intraperitoneally or orally (50 or 150 mg/kg) in combination with the chemotherapeutic agent epirubicin or radiotherapy. The combination of propolis and chemotherapy significantly decreased the lung metastatic rate in comparison with the effects of epirubicin or propolis alone. Moreover, propolis increased the lifespan of mice that received the radiotherapy with gamma rays.

Quercetin, yet another important and well-investigated compound from propolis, similar to caffeic acid, CAPE, and other propolis-related molecules, displays proapoptotic, anti-invasive, and antiangiogenic properties [367,368,369,370,371,372,373,374,375]. Epidemiological studies have indicated that supplementation with quercetin may be associated with the prevention of lung, breast, colon, ovarian, and prostate cancer [367,368,369,370,371,374,375]. Quercetin decreased the invasion of the highly metastatic B16-BL6 melanoma cells in vitro [376]. Furthermore, in combination with resveratrol and catechins, quercetin reduced the distal metastatic invasions in nude mice, especially to liver and bone, by upregulating the expression of Forkhead Box O1 (*FOXO1*) and *NFKBIA* (NF-κB Inhibitor Alpha) genes, which ultimately activated apoptosis and inhibited NF-κB activity [377]. Quercetin also induced a slight, although statistically not significant, reduction in lung metastasis of murine colon cancer cells [368]. In yet another study, the effect of quercetin on the formation of metastasis was investigated in mice injected *iv* with mammary carcinoma cells. Quercetin was given *po* at a dose of 1200 mg kg^−1^ and was ineffective when given for seven consecutive days before the inoculation of tumor cells. However, continuous treatment with quercetin during 7 days before and 7 days after tumor cell inoculation markedly reduced metastases development. The ability of quercetin to inhibit metastasis formation and induce apoptosis may act in concert with its inhibitory effects on matrix degradation and neovascularization, as evidenced by the downregulation of MMP-2, MMP-9, PLGF, VEGF, and its receptors, and HIF-1*α*, together with the increased expression of tissue inhibitor of metalloproteinases 2 (TIMP-2) and reversion-inducing cysteine-rich protein with Kazal motifs (RECK) [378,379,380]. Other studies also indicated that quercetin and its glycosides may suppress the expression of MMPs, upregulate TIMP-2 levels, and reduce VEGF production and the activation of HIF-1*α* [156,229,378,379]. The antiangiogenic activity of quercetin has been assigned to its ATP-mimetic properties and an ability to modulate activation and nuclear import of HIF-1*α* [379]. Inhibition of HDAC-1 and DNMT1 is yet another important activity of quercetin when considering the therapeutic reversal and/or gene silencing by HDAC and DNMT inhibitors [380].

The anti-metastatic potential of chrysin is also well documented. Thus, it was found that chrysin inhibits VEGF production and hypoxia-induced STAT3 phosphorylation without affecting the expression of HIF-1α. Chrysin also abolished hypoxia-induced *VEGF* gene expression [381,382,383]. The authors further found that chrysin could be useful in controlling metastatic progression as it significantly reduced the size of metastatic colonies [381]. As their total number was only slightly decreased, this probably indicates that chrysin disturbs tumor growth after the arrival of tumor cells at metastatic sites. Considering the important role of angiogenesis in the progression of micrometastases to macrometastases, the authors suggested that chrysin interferes with the angiogenesis in hypoxic micrometastases by suppressing activation of hypoxia-induced STAT3 and VEGF secretion. Chrysin is also able to inhibit lymphangiogenesis in vitro [384].

Some other polyphenols have also proven effective as anti-metastatic agents. Pinocembrin, one of the major water-insoluble flavonoids in propolis, inhibited EMT and metastasis by preventing NF-κB translocation to MMP promoter sites [385,386]. Furthermore, at non-cytotoxic concentrations, pinocembrin suppressed the TGF-β1-induced cell-matrix adhesion, invasion, and migration of human retinoblastoma Y-79 cells. It attenuated the TGF-β1-induced expression of vimentin, N-cadherin, and αvβ3 integrin, reduced the expression of MMP-2 and MMP-9, inhibited the activation of focal adhesion kinase (FAK) and phosphorylation of p38α, and decreased nuclear levels and the DNA binding activity of NF-κB and degradation of inhibitor of κBα (IκBα).

Guo et al. [386] demonstrated that genistein could modulate the immune response in adult B6C3F1 mice. They found that genistein exerts chemopreventive effects. It reduced the number of lung tumor nodules following tumor injection and increased host resistance to a B16F10 tumor, probably by increasing the activities of cytotoxic T cells and NK cells. The IL-2-stimulated activity of NK cells was significantly increased with a high dose of genistein [386,387]. In addition, some in vitro studies showed that genistein could directly suppress the proliferation of tumor cells [388]. It should be noted that genistein inhibits tyrosine kinases at high concentrations and acts as an estrogenic compound at low concentrations [388,389]. Zhu et al. [389] demonstrated that genistein reduced the viability of HT29 colon cancer cells and decreased their invasive/migrating abilities. Genistein also downregulated the expression of MMP-2 and MMP-9, but increased the expression of E-cadherin, and recovered activity of Wnt inhibitory factor 1 by modifying the expression of invasion-associated factors, and molecules of the Wnt signaling pathway. In addition, co-administration of genistein and doxorubicin-loaded polypeptide nanoparticles inhibited the distant metastasis of malignant prostate cancer by increasing oxidative injury [390,391].

## 5. Propolis and Its Polyphenolic/Flavonoid Compounds in the Regulation of Glucose Metabolism in Tumor

Tumors and accompanying inflammation and circulatory impairments interfere with the acid–base homeostasis of the body. Unregulated changes in the microenvironment and intracellular pH may affect processes of proliferation, differentiation, metastasis, and angiogenesis. Cancer cells have up to a 17-fold increase in the consumption of glucose than nonmalignant cells, utilizing glycolysis as the predominant metabolic pathway under both normoxia (Warburg effect) and hypoxia [392]. The increased glycolytic flux that is required for rapid proliferation of tumor cells is usually accompanied by the overexpression and/or enhanced activity of specific isoforms of glycolytic enzymes. Glucose transporters and enzymes with a pivotal role in glycolysis, such as hexokinase II (HKII), glyceraldehyde-3-phosphate dehydrogenase (GAPDH), lactate dehydrogenase (LDH), and the isoform M2 of pyruvate kinase (PKM2) are usually upregulated in cancer cells and have been proposed as possible therapeutic targets. Of note, the non-glycolytic activities of several of these enzymes have been described, and both glycolytic and non-glycolytic functions contribute to cancer aggressiveness. For example, GAPDH, LDH, or PKM2 may activate gene expression acting either as direct transcriptional factors or by modulating activity of nuclear proteins, such as HIF-1 and STAT3, which regulate the transcription of genes involved in cell proliferation (e.g., histones H2A and H2B, MEK5, c-Myc, cyclin D1, and androgen receptors) [392,393].

Aerobic glycolysis (Warburg effect) implies the overproduction of protons through several mechanisms. These protons need to be removed as their accumulation results in lethal intracellular acidosis. So, the main source of environmental acidification is the accumulation of protons in the extracellular matrix as by-products of intracellular metabolism. On the other hand, increased proliferation and tumor growth deplete oxygen and other nutrients, leading to hypoxia and upregulation of HIF-1 that further drives the expression of various genes [394].

The acidic environment, together with the acquired adaptive cellular modifications, stimulates migration and invasion, and finally may result in metastasis [395]. Lactate plays particularly important roles in these processes: it activates the glycolytic pathway, stimulates angiogenesis and spreading of cancer cells from the primary site, promotes upregulation of HIF-1, destabilizes the immune system, impedes the function of specific immune cells (including cytotoxic T lymphocytes) and cytokine release, and stimulates cell motility [393,394,395,396,397].

As mentioned, HIF-1 induces the expression of various genes, most of them being related to the low (or null) oxygen consumption/metabolism, and the stimulation of neoangiogenesis through increased HIF-1/VEGF secretion, both in cancer cells and in peritumoral fibroblasts, macrophages, myofibroblasts, and endothelial cells. However, as the newly formed vessels are usually not fully functional [393], the hypoxic environment persists as a permanent feature of the cancer.

Thus, HIF-1 upregulates glucose transporters (GLUT) and glycolytic enzymes, including hexokinase, pyruvate kinase, and LDH. It is the pivotal transcription factor that drives metabolic reprogramming by promoting the transcription of over 100 genes involved in angiogenesis, cell migration, cell survival, and energy metabolism. Apart from HIF-1, other signaling pathways, such as STAT3, Akt, ERK, and NF-κB, are also activated during hypoxia and modulate the expression of the hypoxia-induced *VEGF* gene [393,394,395,396].

For example, the increase in glucose utilization and lactate production observed in MDA-MB-231 cells was explained by overexpression of HIF-1𝛼, pyruvate dehydrogenase kinase, glucose transporter 1, LDH, and carbonic anhydrase. On the other hand, Portuguese propolis disturbed the glycolytic metabolism of human colorectal cancer cells, as evidenced by a decrease in glucose consumption and lactate production [397]. Similarly, Chinese poplar propolis reduced the glycolytic metabolic pathway by inhibiting HK2, phosphofructokinase, the muscle isoenzyme PKM2, and lactate dehydrogenase A (LDHA) in LPS-induced inflammation [398]. Some components of Brazilian green propolis, baccharin and drupanin, can inhibit angiogenesis by inhibiting the expression of HIF-1 and its targets (GLUT1, HKII, and VEGF) [399,400].

Hence, natural compounds are capable of modulating glycolysis in a tumor environment. In Caco-2 colon cancer cells, flavonoids naringenin, morin, silybin, and quercetin competitively inhibited monocarboxylate transporters (MCT1) that function as proton-linked membrane carriers for lactate and pyruvate [80,401]. In addition, apigenin, biochanin A, chrysin, diosemin, fisetin, genistein, hesperitin, kaempferol, luteolin, morin, narigenin, phloretin, and quercetin may modulate pharmacokinetics and pharmacodynamics of MCT1 substrates [402]. Phenolic acids can also be involved in the inhibition of MCT activity. Alpha-cyano-4-hydroxycinnamic acid, a derivative of cinnamic acid, is a competitive inhibitor of lactate transport and has been linked to the inhibition of MCT activity in several tumor models [403,404,405,406,407]. However, further studies are needed to better understand the mechanisms by which propolis affects glycolytic metabolism; in particular, if its effects are mediated by the inhibition of lactate transport via MCTs.

Additionally, propolis and its components such as genistein, phloretin, apigenin, and daidzein, can modify glucose uptake by modulating GLUT1 expression or glucose binding [400]. The inhibition of glucose uptake inhibits cell growth and triggers apoptosis in some tumor cells. Thus, propolis and its components (fisetin, myricetin, quercetin, resveratrol, apigenin, genistein, cyanidin, daidzein, hesperetin, and naringenin), which are capable of inhibiting enzymes involved in glucose metabolism such as GLUT, hexokinase, or PKM2, could represent a valuable option in cancer treatment [82,407,408,409,410,411,412,413,414].

Gonzalez-Menendez [407] compared the effects of four flavonoids on androgen-sensitive and -insensitive prostate cancer cells that differ in GLUT expression and glucose uptake. They found that the effects of flavonoids were dependent on cellular phenotype and that flavonoids with the highest antiproliferative effect (apigenin and phloretin) were also the most effective in decreasing glucose uptake and GLUT expression. Similarly, the effects of resveratrol on viability and glucose uptake have been investigated in human ovarian cancer cell lines and in vivo mice models. Resveratrol reduced glucose uptake, lactate production, signaling through the Akt and mTOR pathways, and cell viability [410,411]. Furthermore, a resveratrol-induced decrease in glucose uptake has been observed in mouse Lewis lung carcinoma cells (3LL) and was dependent on its anti-oxidant properties. Further studies in two human leukemic cell lines (U-937, HL-60) and in human erythrocytes indicated that resveratrol can suppress GLUT1-mediated transport and hexokinase-mediated glucose trapping [82,414].

Finally, it should be emphasized that various natural inhibitors of glycolytic enzymes from propolis may enhance therapeutic efficacy and help overcome drug resistance in multiple cancer cells [317]. In addition, ATP depletion as a result of glycolysis inhibition may promote intracellular drug accumulation, leading to increased drug sensitization [402].

## 6. Telomerase-Related Anticancer Strategies by Propolis and Its Polyphenolic/Flavonoid Components

Telomerase is an enzyme mainly involved in the maintenance of telomere length but is also considered as an important factor in almost all cancer cells. Telomerase enables infinite cell proliferation during malignancy. Enhanced telomerase activity is positively correlated with deficient differentiation and increased mortality rate in patients with adenocarcinoma and small cell lung carcinoma [415,416,417]. Similarly, high levels of telomerase are found in breast cancer patients [418], colorectal cancers [419], and gastrointestinal cancer [69]. A catalytic subunit of human telomerase is telomerase reverse transcriptase (hTERT), and telomerase expression in most cancer cells is proportional to the expression of hTERT mRNA [420,421]. Accordingly, telomerase inactivation or its destabilization, in particular by natural products such as propolis and its polyphenolic constituents, is considered to be a promising approach for drug development in cancer therapy as progressive telomere shortening to a critical level results in apoptosis.

In leukemia cells, propolis successfully reduced hTERT mRNA expression [64]. Thus, hTERT expression in a T-cell acute lymphoblastic leukemia cell line treated with Manisa propolis decreased from 195.56 to 13.29 in 72 h, whereas cell viability was not affected [422]. Similarly, in leukemia cells obtained from the bone marrow of pediatric patients (three acute lymphoblastic leukemia and one chronic myeloid leukemia), treatment with ethanolic extract of Manisa propolis depleted the expression of hTERT [423].

Numerous polyphenolic components from propolis show the inhibition of telomerase activity, including resveratrol, genistein, quercetin, curcumin, apigenin, daidzein, gallic acid, ellagic acid, luteolin-7-0-glucoside, etc., which may add new therapeutic value to cancer treatment by inhibiting hTERT and hTR, telomerase substrates, and their associated proteins [64,423,424,425].

Similar studies have confirmed the inhibitory effect of resveratrol (3,5,40-trihydroxy-trans-stilbene) and pterostilbene, a natural analog of resveratrol, on telomerase activity, together with the down-regulation of hTERT expression in various cancer cell lines [68,426,427], which ultimately induced senescence through the DNA damage response. Tannic acid also reduced telomerase activity, cell viability, and cell count in human breast (MCF-7) and human colon cancer (CaCo-2) cell lines [428,429]. Likewise, quercetin reduced telomerase activity, down-regulated hTERT expression, and induced apoptosis, thus preventing the growth of various cancer cell lines (lung, stomach, colon, nasopharyngeal, laryngeal, and breast) [69,428,429].

## 7. Present Status and Future Perspectives

In conclusion, this review highlights the therapeutic potential of propolis and its polyphenolic/flavonoid compounds against cancer. Antitumorigenic properties of these attractive natural products and functional ingredients rely on powerful antioxidant and anti-inflammatory effects, regulation of the cell cycle arrest and proliferation, apoptosis/autophagy, angiogenesis, invasions, metastasis spreading processes, and the ability to regulate epigenetic mechanisms and blood glucose levels. Results presented here indicate that honeybee propolis and its polyphenolic components might be useful adjuvants in the control of tumor growth in vitro and in vivo. The potential anticancer effects of propolis and its polyphenolic/flavonoid compounds can be summarized into the following cellular and molecular mechanisms of action: (i) suppression of cancer/precancerous cell proliferation via direct cytotoxic effect or via its immunomodulatory effect; (ii) reduction in cancer stem cell populations; (iii) inhibition of the specific oncogene signaling pathways; (iv) antiangiogenic effects; (v) modulation of the tumor microenvironment; (vi) inhibition of the cellular glucose uptake and metabolism in the cancer cell, and finally; (vii) as a supplementary or complementary approach to conventional anticancer therapies. Current research shows that propolis and its components inhibit multiple signaling pathways essential for the initiation, progression, and metastasis of cancer, such as PI3K/AKT/mTOR, NFκB, JAK-STAT, TLR4, VEGF, TGFβ, and apoptosis and autophagy pathways. The therapeutic effects of propolis extracts are mostly assigned to its active compounds, such as CAPE, quercetin, hesperidin; and artepillin C, which are capable of activating macrophages and produce factors involved in the regulation of B-, T-, and NK-cell functions. These natural ingredients of propolis induce a prominent reduction in CD4 + CD25 + FoxP3+ regulatory T cells that lead to suppression of IL-10 or TGF-β production and antitumor activity. Moreover, inhibition of the M2 phenotype and stimulation of the M1 macrophage polarization associated with the production of Th1 cytokines such as TNF-α, IFN-γ, and colony-stimulating factor 2 (CSF2), increases antigen-presenting capacity, as well as the production of ROS and cytokines (IL-6, IL-12, IL-23, and TNF-α) that both contribute to microbicidal and pro-inflammatory activities. M1 macrophages are termed as “fighting” macrophages and are associated with a good prognosis. Reprogramming M2 TAMs toward a more pro-inflammatory M1 phenotype may be an important parameter for clinical application given that the ratio of M1/M2 macrophages is involved in immunoediting, angiogenesis and neovascularization, stromal orchestration, and response to therapy. The immunomodulatory effect of propolis and flavonoids in the stimulation of IFN-γ production can be important in strengthening specific and non-specific immunity, especially in the activation of macrophages, NK, and other cells, and is also used as a mechanism in gene therapy. Accordingly, due to TAMs localization in the tumor hypoxia regions, viral vectors can be used to transduce macrophages with therapeutic genes, such as IFNc, which are activated only in low oxygen conditions. This strategy can be a promising approach that uses macrophages as vehicles to deliver gene therapy to hypoxic regions in tumors.

In addition, propolis and its polyphenols possess multiple cell-regulatory activities. Their chemopreventive effects are, at least partially, related to the modulation of various components of the epigenetic machinery in humans and animals. As epigenetic modifications are potentially reversible, they represent a reasonable approach in cancer prevention and therapeutic strategies. Considering the impact of acetylation on tumor metabolism and the effect of polyphenolic components of propolis on the modulation of epigenetic activity, their effect on metabolic changes should be investigated further. Future research should be focused on the mechanisms of epigenetic modifications induced by propolis and its components, and the possibility of their utilization in the development of novel pharmacologic approaches directed toward efficient preventive and chemopreventive agents against cancer.

Cancer cell energy metabolism is an important target for the improvement of therapeutic strategies. Cancer cells possess a 20- to 30-fold increased rate of cellular glucose uptake and a more than 30-fold higher glycolytic rate. This increased dependence of cancer cells on extracellular glucose levels is necessary to support a high rate of glycolysis that interferes with cellular glucose uptake and makes glycolysis an attractive anticancer target. In that regard, polyphenols may bring an important advancement in anticancer therapy.

Deregulation of apoptotic cell death machinery is yet another hallmark of cancer. Failure of apoptosis is responsible not only for tumor development and progression, but also for tumor resistance to therapies. The development of apoptosis-reactivating strategies may improve the efficacy of cancer therapy and bypass resistance. Polyphenolic compounds from propolis induce apoptotic processes by activating Bax, p53, and p21 proteins, p38, JNK, and ERK MAP kinases, and the release of cytochrome c, triggering the caspase cascade. In addition, these components through the inhibition of NF-κB suppress important anti-apoptotic proteins such as IAP, c-FLIP, and Akt kinase, and initiate the extrinsic pathway of apoptosis by inducing TRAIL and Fas receptor stimulation in cancer cells. Importantly, these effects are not related to a specific type of tumor cells and are initiated in different types of cancers through the interactions with the multiple proteins in various cell signaling pathways. Thus, propolis and its flavonoids may act on pathways involved in the prevention of metastatic progression, inhibition of NF-κB translocation, modulation of gene expression, inactivation of MMPs, and induction of tumor suppressors, acting as histone deacetylase inhibitors for epigenetic therapy and overcoming the TRAIL resistance of cancer cells.

Propolis also exerts its protective effect against different viral and bacterial pathogens that are potential carcinogens, by acting on both host and bacterial physiology at different enzymatic and immunological cascade reaction steps. Since many conventional treatments still fail to achieve the complete eradication of viral and bacterial pathogens, such as *H. pylori*, and possess a high risk of developing microbial resistance, natural products are useful alternatives, either as a main-stream treatment or as an adjuvant. Although it has been confirmed that propolis and its flavonoid components can inhibit the growth of *H. pylori*, additional microbiological research is needed before the potential clinical application of these natural products.

Although there are a large number of standardized preparations of propolis extracts available, they are usually used to treat herpes lesions or as preparations in the wound healing process (EPP-AF^®^ and PE-8, Brazilian green propolis; GH 2002, propolis from the Czech Republic, PropoelixTM and M.E.D.^®^, standardized extract of poplar propolis; PP-Lip, propolis liposomes, and other preparations) (see review [430]), the major limitation of the potential use of propolis in the clinic is the exact determination of its components and standardization according to biologically active polyphenolic/flavonoid constituents that are essential for the inhibition of the development, progression, angiogenesis, and metastasis of tumors. Its diversity is very high and depends on a number of factors, including geographical area, climate, plant species, method of storage and collection, storage time, and the type of bees in the bee community. The wide variability of polyphenolic/flavonoid components, despite the confirmed synergism in some tumor stages, could have an additive and/or antagonistic effect. Furthermore, different pesticides or heavy metals can be found in propolis, which can cause unwanted side effects. In addition, some constituents of propolis can cause allergic reactions in some patients. Such allergenic components should be removed from propolis for patients prone to the development of an allergic reaction. The most common allergenic components of propolis are 3-methyl-2-butenyl caffeate, phenylethyl caffeate, benzyl caffeate, geranyl caffeate, benzyl alcohol benzyl cinnammate, methyl cinnammate, ferulic acid, and tecto chrysin [38,43]. Some of the observed symptoms of allergic reactions are contact chelitis, contact stomatitis, perioral eczema, labial edema, oral pain, peeling of lips, and dyspnea.

Furthermore, the question arises about the tumor variability (at least 200 types), different signaling pathways and redox status, and the stages of tumor development and different possibilities of bioavailability of propolis and its components. It is known that numerous signaling pathways and processes stimulated by propolis and flavonoids can be either positive in the early phase of tumor formation (initiation process) or negative in highly advanced tumors. For example, autophagy induced in apoptosis-resistant tumors can be important in the prevention of tumor formation, but it can be negative in an advanced stage of the tumor and enable the survival of tumor cells and the development of resistant cells to various forms of therapy, including chemotherapy and radiotherapy. Moreover, activation of the Nrf2 signaling pathway by propolis and flavonoids in the late phase of tumor development can contribute to resistance to chemo- and radiotherapy [127,140,141,142,241,250]. Furthermore, different sensitivity of tumor cells was confirmed depending on the different stages of tumor development. Certainly, the different sensitivity of tumor cells to the oxidation–reduction status of flavonoids, the possibility of antioxidant/pro-oxidative action, as well as action on hormone receptors, should be better investigated. Future research should be directed into the possibilities of individual flavonoids and/or combinations of individual flavonoid components as well as propolis fractions in order to better utilize these possibilities in the prevention and therapy of tumors. It is known that tumors usually accumulate metals more than normal cells. This could be a good approach in the local application of flavonoids, production of high levels of ROS, and induction of cell death, for example, by ferroptosis. Certainly, glucose metabolism of tumor cells, changes in pH, active channels and ATP pumps, and epigenetic regulation, are important mechanisms that can be used as a target in the action of propolis and flavonoids. It should also be pointed out that different polyphenolic components and their structural diversity, can have different effects on the intestinal microbiota and their enzymatic activity, which affects the bioavailability, metabolism, and bioactivity of flavonoids, the diversity of which can be increased depending on the patient’s age, health, and polymorphism of CYP enzymes.

In addition to the above, a part of the limitations can be based on the limited use of animal models and the diversity of tumors, as well as on numerous physiological and biochemical differences between animal models and humans. Experimental data obtained in rats are not directly translatable to humans. With the exception of the dose corrected by the body surface area, many other parameters such as oxygen utilization, caloric expenditure, basal metabolism, blood volume, circulating proteins in plasma, and renal function should be considered. Additional limitations include anatomical differences, differences in genetic regulation, different pathophysiological mechanisms, different drug responses, differences between species, and differences caused by the route of application.

Finally, it must be emphasized that natural chemicals have gained substantial attention in cancer therapy due to their favorable safety profile (low toxicities) and ability to trigger multiple signal transduction pathways. Unfortunately, the antitumor effects of natural products alone are far from satisfactory. Due to the low bioavailability and clinical efficacy of propolis and its flavonoids, their biomedical applications remain limited. The poor physicochemical properties of most flavonoids have forced many researchers into the development of nanoformulations to increase the bioavailability of these phytochemicals. Polymeric nanocapsules, nanomicelles, liposomes, nanodiamonds, as well as various other nanoformulations, have been employed to enhance the bioavailability, and ultimately, the protective and anticancer properties of flavonoids. In addition, highly efficient and selective drug delivery is important for improvement in the current therapeutic regimens that will increase the activity on cancer cells and reduce toxicity to normal cells. The use of nanoparticulate drug carriers may help to resolve current challenges in drug delivery to the cancer cells, including improvement in drug solubility and stability, extending the half-life of anticancer agents in the blood, reduction in the adverse effects in non-target organs, and aiding in the delivery of high concentrations of anticancer drugs to the site of the disease, providing more effective treatments and better results. Hopefully, propolis can become an attractive and promising agent for cancer prevention and treatment.

## Figures and Tables

**Table 1:** The major types of propolis, their geographical origin, and major constituents.

Propolis Type	Geographic Origin	Plant Source	Typical Chemical Constituents
Poplar propolis	Europe, North America, non-tropical regions of Asia	*Populus* spp. (most often *P. nigra* L.)	pinocembrin, pinobanksin, pinobanksin-3-O-acetate, chrysin, galangin, phenolic acids, and their esters
Birch propolis	Russia	*Betula verucosa* Ehrh.	acacetin, apigenin, ermanin, rhamnocitrin, kaempferid, α-acetoxybetulenol, cinnamic acids, phenylpropanoid sesquiterpenols,
Green (Alecrim) propolis	Brazil	*Baccharis* ssp. (most often *B. dracunculifolia* DC.)	prenylated p-coumaric acids and o-hydroxy-acetophenone, labdane, diterpenic acids
Red propolis	Cuba, Mexico, Brazil, Venezuela, Amazon	*Clussia* ssp.(?) *Clusia* flower *Dalbergia ecastaphyllum*	phenylpropene derivative elemicin, triterpenic alcohol β-amyrin, prenylated benzophenones, polyprenylated benzophenones, formononetin, isoliquiritigenin, liquiritigenin, medicarpin, and biochanin A
Mediterranean propolis	Greece, Malta, Crete, Cyprus, Turkey, Algeria, Southern Italy	*Cupressaceae*/*Juniperus*/*Pinus* family), *Conifer* spp., *Ferula communis*, *Castanea sativa*, *Cistus* spp., *Quercus ilex* L., *Fraxinus ornus* L., and *Olea europaea* L.	diterpenes, communic, cupressic and isocupressic acids, totarol, labdane, abietane diterpenes, clerodane, pinobanksin esters, anthraquinones, esters of caffeic and ferulic acids,
“Canarian” propolis	Canarian Islands	unknown	furoruran lignans (sesamin, episesamin, methyl xanthoxylol, aschantin, sesartenin, and yangambin), sesquiterpenoids, spatulenol, nerolidol
“Pacific” propolis	Okinawa, Taiwan, Japan	*Macaranga* plants, *Macaranga tanarius*	C-prenyl-flavanones prenylflavonoids, more specifically isonymphaeol-B, nymphaeol-A, nymphaeol-B, nymphaeol-C, propolins, 3′-geranyl-naringenin

**Table 2:** Stages of carcinogenesis inhibited by propolis and its polyphenolics/flavonoids and the possible molecular mechanisms of their biological actions.

Stages of Carcinogenesis	Mechanisms of Carcinogenesis	Action of Dietary Polyphenolics/Flavonoid Components from Propolis:
**1. Initiation stage**	**Mutagens, activation of carcinogens** **⇒ DNA damage** **⇒ mutations**	**Antimutagenic effect** **Carcinogen metabolism** ↓ Phase I metabolizing enzymes (CYP1A activity) - Transformation of xenobiotics by oxidation, reduction, or hydrolysis ↑ Phase II metabolizing enzymes (detoxification) - Conjugation of xenobiotics and their metabolites to endogenous molecules. The net result is a formation of derivatives that are more hydrophilic and easily excreted in urine, or (via the bile) in the feces.
	**Epigenetic** **modifications**	**Demethylation, re-expression of TSGs, HDAC inhibition, regulation of expression of non-coding miRNA** ↓ DNMT, HDAC ↑ HAT - Flavonoids modify level of miRNAs via several mechanisms, such as epigenetic changes, transcriptional changes, and either increased or decreased levels of miRNAs (miRNAs with oncogenic functions, called oncomirs, if the target mRNA is a tumor suppressor gene (Let-7, miR-15, miR-16, miR-34, miR-45, miR-126, miR-150, miR-183, miR-203, miR-206, miR-335, miR-495) or with tumor suppressor functions, tumor-suppressor miRNAs, if the target molecule is an oncogene (miR-21, miR-155, miR-221, miR-222, miR-373, miR-424-5p, miR-520c)
**2. Promotion stage**	**Tumor promotors** - ROS - Mediators of inflammation - Growth factors - Hormones	**Antioxidative mechanisms** ↑ Scavenging of OH^●^, hydrogen peroxide (H_2_O_2_), superoxide anions (O_2_^−●^), perhydroxyl radical (HO_2_^●^), ROO^●^ ↑ Gap-junctional intracellular communication **Anti-inflammatory mechanisms** ↓ Prooxidant enzymes (xanthine oxidase (XO), cyclooxygenase (COX), lipoxygenase (LOX)). ↓ NF-κB
**3. Tumor progression stage**	**Uncontrolled cell growth**	**Signal transduction pathways** ↓EGF/EGFR signaling ↓GSK3β (Wnt-signaling) ↓Polyamine metabolism (PKC, ODC) **Inhibition of cell growth** ↓Notch, Wnt, PI3K/Akt, NF-κB, AP-1 ↑p53, p21Cip1/waf1, p27 ↑cyclins-d1, E, B - inhibition of different protein kinases involved in the regulation of cell proliferation, differentiation, and transformation - inhibition of DNA synthesis enzymes (ribonucleotide reductase, DNA polymerase, or topoisomerase II) **Induction of apoptosis** - ↓Bcl_2_, XIAP, survivin - ↑Bax, Bid, Cyt C, caspase activation, FOXO1 - inhibition of heat shock proteins - topoisomerase-mediated apoptosis - mitochondrial toxin-mediated apoptosis by activation of Bax, p53, p21 proteins, MAPK kinases—p38, JNK, and ERK, mitochondrial cytochrome c release, and caspase activation - tumor necrosis factor-related apoptosis-inducing ligand (TRAIL) or Fas receptors - inhibition of antiapoptotic proteins (Akt, IAP, c-FLIP) - oxidative stress-induced apoptosis - other mechanisms for inducing apoptosis
	**Angiogenesis and Metastasis**	**↑** **Innate immunity** **↑Cell-cell attachment** **↓Cell migration** **↓Cell-matrix adhesion** **↓Angiogenesis** - **↓**EGFR, VEGFR, TGF-α, PDGGF, VEGF, FAK, Src - **↓**EMT-inducing transcription factors (snail, Zeb, and/or twist)

**Table 3:** Potential tumor preventive mechanism(s) of propolis and related flavonoids.

Mechanisms	Effectiveness Based On:
**Antioxidant activity**	***Antioxidative effect of flavonoids in biological system is related to:*** - ability to scavenge ROS including singlet oxygen (^1^ O_2_), hydroxyl radical (OH^●^), hydrogen peroxide (H_2_O_2_), superoxide anions (O_2_^−●^), perhydroxy radical (HO_2_^●^), lipid radical (LO^●^), and lipid peroxy radical (LOO^●^) - ability to scavenge nitric reactive radical (HOONO, NO, NO_3,_ and others) - suppression of oxidative enzymes; - chelation of metal ions (Fe^2+^, Cu^2+^, Zn^2+^ and, Mg^2+^) - increased activity and protection of antioxidant enzymes - synergistic action with other antioxidants - inhibition of Nrf2 degradation and increase in transcriptional activity of protective genes such as antioxidant proteins and phase II detoxification enzymes
**Inhibition of** **nitrosation and** **nitration**	- flavonoids directly react with ONOO^−^ or scavenge ·OH and ·NO_2_ generated by ONOO^−^, thus blocking the nitration reaction - peroxynitrite scavenging and further inhibition of tyrosine nitration, DNA strand breaks, and oxidation of low-density lipoproteins
**Reduction in** **iron ions**	***Iron reduction has multiple anticancer actions*, *including:*** - depriving neoplastic cells of a key required nutrient, - producing an antiangiogenic effect due to decreased ferritin - inhibition of the formation of 8-hydroxydeoxyguanosine in vivo - influencing cell cycle regulation at multiple sites - tumor suppressor genes may have specific vulnerability to the iron-catalyzed Fenton reaction - decreasing prooxidant activity of iron and other metal ions
**Anti-inflammatory** **effects**	***Anti-inflammatory effect of flavonoids is related to*:** Antioxidant activity - radical scavenging - inhibition of ROS production - inhibition of prooxidative enzymes Regulation of inflammatory cells - modification of enzymatic activity - adjustment of secretory processes Regulation of proinflammatory enzymes - suppression of arachidonic acid enzymes - NO synthase inhibition Modulation of proinflammatory mediators - modulation of cytokine and chemokines production - modulation of MMP Modulation of pro-inflammatory gene expression - modulation of signal transduction Inhibition of the Toll-like receptor 4 (TLR-4) signaling pathway
**Antimutagenic mechanisms**	***Anti-mutagenic effect of flavonoids is related to*:** ***Extracellular mechanisms*** - inhibition of mutagen uptake - inhibition of endogenous mutagens (inhibition of nitrosation; modification of the intestinal flora) - formation of complexes with mutagens and/or their deactivation - preferable absorption of protective compounds ***Cellular mechanisms*** - inhibition of or competition with mutagens (ROS scavenging; protection of DNA nucleophilic sites) - trapping and detoxification in non-target cells - modification of transmembrane transport - altered function of xenobiotic metabolizing enzymes (inhibition of promutagen activation; activation of detoxification pathways) - modulation of DNA metabolism and repair - enhancement of apoptosis - maintenance of genomic stability
**Process of** **detoxification** **by fibers**	- increased speed of movement of feces through colon - dilutes carcinogens and/or slows their formation
**Enzyme** **inhibition**	***Enzyme inhibition effect of flavonoids is related to*:** - cyclooxygenase-2 - inducible nitric oxide synthase - xanthine oxidase - phase I enzyme (P450 enzymes, block activation of carcinogens; CYP1A1, CYP1A2, CYP1B1, CYP2E1, CYP3A4, CYP19)
**Enzyme** **induction and** **enhanced** **detoxification**	***Enzymes included in this reaction are:*** - sulfotransferases (SULT1A1, SULT1A3, SULT1E1) - UDP-glucuronosyltransferases (UGT, UGT1A1) - quinone reductase (QR) - acetyltransferases ***Effect is related to:*** - inhibition of organic anion transporters participating in the uptake of nephrotoxic compounds by flavonoids and their phase II metabolites (sulfates, glucuronides) - xenobiotic uptake and efflux by solute carrier transporters; organic anion transporting polypeptides (OATPs), organic anion and cation transporters (OATs and OCTs, respectively) and ATP-binding cassette (ABC) transporters
**Inhibition of** **signal transduction** **pathways**	***Regulate the signal transduction pathways including:*** - NF-κB signaling - PI3K/Akt/mTOR signaling - Hedgehog signaling - Akt, MAPKs, p53 - androgen receptor (AR), and estrogen receptor (ER) pathways - STAT signaling - AP-1 signaling - Notch-1 signaling - Wnt/β catenin signaling - Insulin-like growth factor (IGF) signaling - NF-E2-related factor 2/antioxidant response element (Nrf2/ARE) pathway
**Inhibition of** **cell** **proliferation**	***Suppression of cancer stem cells self-renewal*, *progenitor formation*, *and clonal growth*** ***Telomerase inhibition*** - by decreasing levels of telomerase reverse transcriptase (hTERT) and hTR, telomerase substrates, and their associated proteins ***The disruption of microtubules in mitosis*** - by down-regulating tubulin in microtubules ***Inhibition of topoisomerase I and II*** ***Proteasome inhibition*** ***Cell cycle inhibition*** - by suppressing expression of cyclin A, cyclin B, Cdk2 - by enhancing the p21 and p27 levels - by inhibiting activity of hTERT in tumor cells ***Inactivation of prooxidative enzymes*** - inhibiting cyclooxygenases and lipoxygenases - decreasing xanthine oxidase ***Inhibition of ornithine decarboxylase and polyamines synthesis*** ***Modulation of signal transduction pathways*** Inhibition of various protein kinases: - protein tyrosine kinase (PTK), - cAMP-dependent protein kinase (PKA), - phosphoinositide 3-kinase (PI3K), - protein kinase C (PKC), - mitogen-activating protein kinases (MAPK), - cyclin-dependent kinases (CDKs), - p34^cdcz^ kinase - focal adhesion kinase (FAK) ***Inhibition of DNA synthesis enzymes*** - ribonucleotide reductase, - DNA polymerase or topoisomerase II
**Induction of cell** **differentiation**	- downregulation of c-Myc, inhibiting the protein kinases
**Inhibition of** **oncogene expression**	- downregulation of c-Jun, c-Fos, c-Myc, Ki-ras
**Induction of tumor** **suppressor gene** **expression**	- modulation of p53, Rh, Bcl-2, p21, p27, BRCA1, BRCA2, RhoB
**Induction of** **cell-cycle arrest**	- quercetin exerts growth inhibitory effect on human colon cancer via related 17 kDa protein, which blocks cell transition from G_0_/G_1_ into the S phase of the cycle - curcumin induces cell cycle arrest in various cell types, preferentially in G_2_/M phase
**Induction of** **apoptosis**	***By inhibition of heat shock proteins*** ***Topoisomerase-mediated apoptosis*** - ATP binding domain of topoisomerase II may serve as the binding site for flavonoids resulting in the inhibition of the ATPase component of the topoisomerization reaction. ***Mitochondrial toxin-mediated apoptosis*** ***Oxidative stress-induced apoptosis*** - rate of autooxidation of flavonoids at pH 7.4 to form H_2_O_2_ (pyrogallol-type flavonoids generate H_2_O_2_) ***Other mechanisms for inducing apoptosis*** - upregulation of Bax and p21 - elevation of intracellular cAMP to high levels with cAMP analogs, adenylate cyclase activators, or phosphodiesterase inhibitors. - increase in Ca^2+^ levels (endonuclease activation) and wild-type p53 overexpression. - upregulation of TRAIL-R1 and TRAIL-R2 - downregulation of p53 and antiapoptotic proteins (Bcl2, IAP, c-FLIP, Akt)
**Enhancement of** **immune functions** **and surveillance**	- increased activity of macrophages, B, T, and NK cells - upregulation of toll-like receptors (TLR2 and TLR4) - inhibition of M1 to M2 macrophage polarization - triggering Immunogenic cell death effect (ICD) - downregulation of the PD-L1 expression through JAK-STAT and NF-κB pathways
**Antiangiogenesis**	- inhibition of EGF, TGFα, bFGF, VEGF, VEGFR, MMPs, claudin, β-catenin, COX2
**Overcoming** **resistance** **to cancer therapy**	- inhibition of P-gp (transcriptional downregulation of MDR-1; direct high-affinity binding to nucleotide-binding domain (NBD); ATPase inhibition; nucleotide hydrolysis; energy-dependent drug interactions with membranes enriched in transporters) - inhibition of CYPs - induction of apoptosis - inhibition of NF-κB - inhibition of glycolysis by inhibitors of glycolytic enzymes
**Interaction with cellular drug transport systems**	- inhibition of P-gp pump, MRP1, BCRP - competition with glucose for transmembrane transport
**Inhibition of** **NF-κB**	***Inhibition of NF-κB leads to negative effects:*** - viability (Survivin, Bcl-2, Bcl-xL, c-FLIP, c-IAP, XIAP) - proliferation (Cyclin D1, CDK, c-Myc, COX-2, IL-1, IL-6, TNF) - invasion (ELAM-1, ICAM-1, MMP, urokinase-type plasminogen activator (u-PA), VCAM-1) - angiogenesis (angiopoietin, VEGF) - metastasis (CXCR4)
**Inhibition of** **cell adhesion** **and invasion**	- by inhibiting matrix metalloproteinase (MMP2, MMP9) - by inhibiting intercellular adhesion molecule (ICAM; mainly ICAM-1) - by inhibiting cysteine proteases and serine proteases such as u-PA and u-PAR - by stimulating cell-cell communication in transformed cells, decreasing malignancy - modulation of the platelet function - downregulation of Rac1, CXCR4, HIF-1α - inhibition of NF-κB and Akt signaling pathways
**Inhibitors of** **metastasis**	- altered expression of oncogenes and tumor suppressors - reduced migration and adhesion of cells - reduced adhesion to laminin substrate and ability of invasion or migration - suppression of cells migration by downregulating cell motility-related genes Rac1 and VASP - EMMPRIN reduction via the PTEN/Akt/HIF-1α signaling pathway - reduced cell motility due to downregulation of MMP-2 and MMP-9 - upregulation of tissue inhibitor of metalloproteinase - inhibition of hypoxia-inducible genes involved in invasion/migration, such as uPAR, ADM, and MMP2 - decreased expression of CXCR4, through the NF-κB suppression - inhibition of TNF-α-induced migration and EMT through Akt/NF-κB pathway inhibition - inhibition of M1 to M2 macrophage polarization - inhibition of EMT-inducing transcription factors (Snail, Zeba, and/or Twist)
**Disruption of** **tumor cell** **glycolytic** **metabolism**	- reduction in glucose consumption and lactate production - inhibition of expression of HIF-1 and its target genes (GLUT1, HKII, and VEGF) - modulation of the expression of glucose transporters (especially GLUT1 and GLUT4) - inhibition of glycolysis by inhibitors of glycolytic enzymes hexokinase 2 (HK2), phosphofructokinase (PFK), muscle isozyme pyruvate kinase M2, (PKM2), and lactate dehydrogenase A (LDHA) - inhibition of lactate transport through monocarboxylate transporters (MCTs)
**Receptor binding**	***High concentrations could modulate receptor or enzyme activity in vivo:*** - by binding to the aryl hydrocarbon receptor and reduction in dioxin toxicity - following binding to estrogen receptor isoflavones and lignans act as phytoestrogens
**Prevention of** **DNA adduct** **formation or** **DNA intercalation**	- free radicals scavenging - protection against DNA adduct formation - protection against chromosome aberration - inhibition of topoisomerase enzymes (accumulation of DNA breaks and mutations without covalent binding to DNA) - prevention of carcinogenesis by N-nitrosamines due to increased glutathione-S-transferase - protection against H_2_O_2_-induced DNA damage by inhibiting DNA strand breaks - protection against 8-hydroxy-2′-deoxyguanosine (8-OHdG) generation and downregulation of nuclear phospho histone H2AX expression - protection against high glucose-induced DNA fragmentation, chromatin condensation, and hypodiploid DNA - protection against oxidative DNA damage (intercalation into the DNA duplex and reaction with free radicals) - helical stabilization by low flavonoid concentration - helix opening by high flavonoid concentration - interaction with telomerase sequences and stabilization of the G-quadruplex structure - reactivation of p53 and DNA repair
**Regulation of** **steroid** **hormone** **metabolism**	- binding to the androgen receptor (AR) and estrogen receptor - prevention of estrogen action in promoting growth of certain tumors - decrease in estrogen biosynthesis through aromatase inhibition
**Effects on** **biomarkers in** **tumor promotion**	- reduction in ROS, xanthine oxidase, NADPH oxidase, and peroxidase - reduction in PKC, calcium release, and calcium canal activation - reduction in ornithine decarboxylase, and polyamine biosynthesis - reduction in cyclooxygenase I and II, arachidonic acid metabolism, prostaglandins, and thromboxanes - reduction in MAPK cascades - inhibition of AP-1 (TPA-response element) and NFκB (IκB kinase, PKC, ROS) - downregulation of c-Jun, c-Fos, c-Myc, Bax, and Cdks (inhibiting oncogene activation) - modulation of p53, Rh, Bcl-2, p21, p27
**Prooxidative effect**	***Prooxidative effect of flavonoids is related to:*** - in the presence of O_2_, transition metals catalyze phenolic redox cycling and formation of reactive oxygen species (ROS) and phenoxyl radicals that can damage DNA, lipids, and other biological targets - autoxidation of flavonols with pyrogallol or catechol B rings in the presence of transition metals increase production of ROS and accelerate oxidation of low-density lipoproteins during the propagation phase - peroxidase-catalyzed oxidation of phenol B ring-containing flavonoids to pro-oxidant phenoxyl radicals - peroxidase-mediated oxidation of catechol B ring-containing flavonoids results in the formation of semiquinone- and quinone-type metabolites - prooxidant phenoxyl radicals cause mitochondrial toxicity
**Antibacterial,** **antiviral, and** **antiparasitic activity**	- reduced tumor formation related to many viruses and bacteria capable to induce inflammation and tumor including: *Epstein*–*Barr virus* (EBV), human immunodeficiency virus (HIV) infection, chronic infection with Hepatitis B (HBV) and C (HCV) virus, human T cell leukemia virus type 1 (HTLV-I) - *Helicobacter pylori* (stomach cancer and MALT-lymphoma) - *Opisthorchis viverrini infection* (bile duct, a rare kind of adenocarcinoma) - *Schisostoma* infection—Schistosomiasis (bladder and colon) - *Herpes simplex virus type 1 and 2* infection (human cervical cancer)
**The main mechanisms** **of antiviral,** **antibacterial, and** **antiparasitic action**	- inhibition of growth and adhesion to mucosal cells - decrease in gastric concentration - enhanced IgA response to the virus - improvement of mucosal barrier function - suppression of proinflammatory cytokine release
**Beneficial effect** **on microbiota** **activity**	***Direct effects of flavonoids are related to:*** - positive effect on microbiota - reduced production of deleterious endotoxins - positive effect on the production of beneficial short-chain fatty acids (SCFA) - systemic effect on glucose homeostasis, lipid, and energy metabolism - prevention of the harmful effects of food, e.g., oxidants and pharmacological insults - inhibition of hydrolytic enzymes, e.g., pancreatic lipase, amylase - alleviation of intestinal permeabilization and associated paracellular transport of endotoxins that can initiate local/systemic inflammation - modulation of the secretion of gut hormones by enteroendocrine cells, which have local effects and systemically modulate energy and metabolic homeostasis - modulation of the GI tract immune system, e.g., Paneth cells (PC) - neutralization of dietary carcinogens ***Indirect effects of microbiota on cancer are related to:*** - beneficial effect of microbiota on inhibition of tumor initiation - beneficial effect of microbiota on prevention and cure of colon cancer - beneficial effect of microbiota on immune system - beneficial effect of microbiota in the protection of the damaged immune system after chemo- and radiotherapy

^1^ Certain mechanisms listed in Table 3 may be involved in several different processes.

## Data Availability

Not applicable.
